# Urate-lowering effect of delphinidin-3-glucoside in red kidney beans via binding to the FAD site of the XO enzyme

**DOI:** 10.1016/j.jare.2025.04.022

**Published:** 2025-04-18

**Authors:** Yanling Chen, Yingtong Jiang, Lei Huang, Ziyi Li, Mengyuan Zhu, Lu Luo, Kun Zhou, Minjian Chen

**Affiliations:** a State Key Laboratory of Reproductive Medicine and Offspring Health, Center for Global Health, School of Public Health, Nanjing Medical University, Nanjing 211166, China; b Key Laboratory of Modern Toxicology of Ministry of Education, School of Public Health, Nanjing Medical University, Nanjing 211166, China; c Department of Epidemiology, Center for Global Health, School of Public Health, Nanjing Medical University, Nanjing 211166, China; d Department of Occupational Medicine and Environmental Health, School of Public Health, Key Laboratory of Public Health Safety and Emergency Prevention and Control Technology of Higher Education Institutions in Jiangsu Province, Nanjing Medical University, Nanjing 211166, China

**Keywords:** Legumes, Hyperuricemia, Delphinidin-3-glucoside, Red kidney beans, XO enzyme

## Abstract

**Background:**

The incidence of hyperuricemia (HUA) is increasing globally, posing serious health risks. The discovery of natural urate-lowering agents is urgently needed.

**Objective:**

To discover natural urate-lowering agents and investigate their effect and action mechanisms for ameliorating HUA.

**Methods:**

Our study comprehensively explored the association between the intake of 13 specific legume varieties in the US population. A composition-target-metabolic (C-T-M) pathway network was constructed to identify key agents and their interactions with key proteins, which were verified by molecular dynamics simulations (MD) and surface plasmon resonance (SPR). Biochemical, *in vitro*, and *in vivo* metabolomic studies in male ICR mice were conducted to examine the effects of the key agent in red kidney beans on uric acid production and other metabolisms.

**Results:**

We found that consuming red kidney beans was robustly negatively associated with the risk of HUA. Based on the C-T-M network, delphinidin-3-glucoside (Dp-3G) was identified as the key agent in red kidney beans, focusing on its binding to xanthine oxidase (XO) enzyme. This interaction was subsequently verified by MD and SPR, revealing that Dp-3G binds to the FAD site of the XO enzyme, thereby blocking electron transfer during enzyme catalysis involving Moco, [2Fe-2S], and FAD. Dp-3G consistently reduces uric acid production under biochemical, *in vitro*, and *in vivo* conditions and reverses metabolic abnormalities related to HUA in mice, including methionine, proline, and folate.

**Conclusions:**

This study identifies Dp-3G, a novel natural agent enriched in red kidney beans, as capable of occupying the FAD site of the XO enzyme, thereby interfering with uric acid synthesis, and suggesting its potential for preventing and treating HUA.

## Highlights

• Red kidney beans reduce the risk of hyperuricemia.
• Delphinidin-3-glucoside is the key bioactive compound in red kidney beans.
• Delphinidin-3-glucoside binds to the FAD site of XO, inhibiting uric acid synthesis.
• Delphinidin-3-glucoside reverses hyperuricemia-related metabolic abnormalities.

## Graphical abstract

**Figure:**
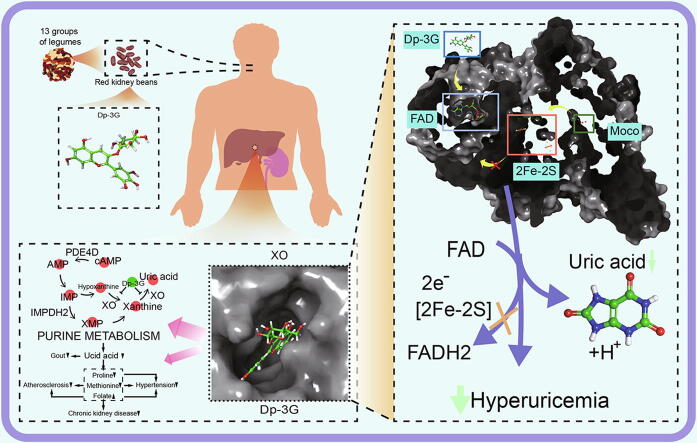

## Introduction

The prevalence of hyperuricemia (HUA) has markedly increased in recent years [1]. HUA is associated with various adverse health outcomes including gout, chronic kidney disease, atherosclerosis, and hypertension [2]. Numerous studies have documented its prevalence globally ranging from 8.4 % to 13.3 % [3]. In the United States, HUA prevalence has risen significantly from 19.1 % (1988–1994) to 21.5 % (2007–2008) [4], and HUA tends to be more prevalent in high-income countries [4]. These findings underscore the critical public health concern posed by abnormal uric acid levels and HUA-related conditions, especially within the US population [5].

Currently, xanthine oxidase inhibitor (XOI) therapy using allopurinol (AP) is recommended as the first-line urate-lowering therapy (ULT). While AP is generally considered safe, approximately 2 % of patients may experience severe hypersensitivity reactions and side effects, occasionally leading to fatalities with a mortality rate of around 20 % [6]. Given their safety profile and potential therapeutic benefits, natural products have become a focus of research aimed at identifying effective preventive or therapeutic agents for HUA.

HUA results from a combination of genetic and environmental factors [7]. Diets rich in purine-containing foods can elevate uric acid levels [7]. Among the dietary factors, the potential impact of legumes on uric acid levels has garnered attention. Legumes, members of the Fabaceae family, encompass a variety of edible seeds from different stages of legume plant maturity: dry beans, peas, lentils, chickpeas, cowpeas, fava beans, and pigeon peas are considered mature legumes. Immature legumes include green beans and peas, while sprouted forms are known as sprouted legumes [8]. Legumes are popular among vegetarians and health-conscious individuals [9]. However, the metabolism of purines within legumes can lead to the production of uric acid. On the contrary, certain compounds in legumes, such as fiber and XOIs may inhibit uric acid formation [10,11]. Due to the diverse compositions of legumes, their specific effects on HUA remain unclear. Exploring novel natural compounds in legumes that may effectively lower uric acid holds promise for future therapeutic developments.

**Fig. 1:**
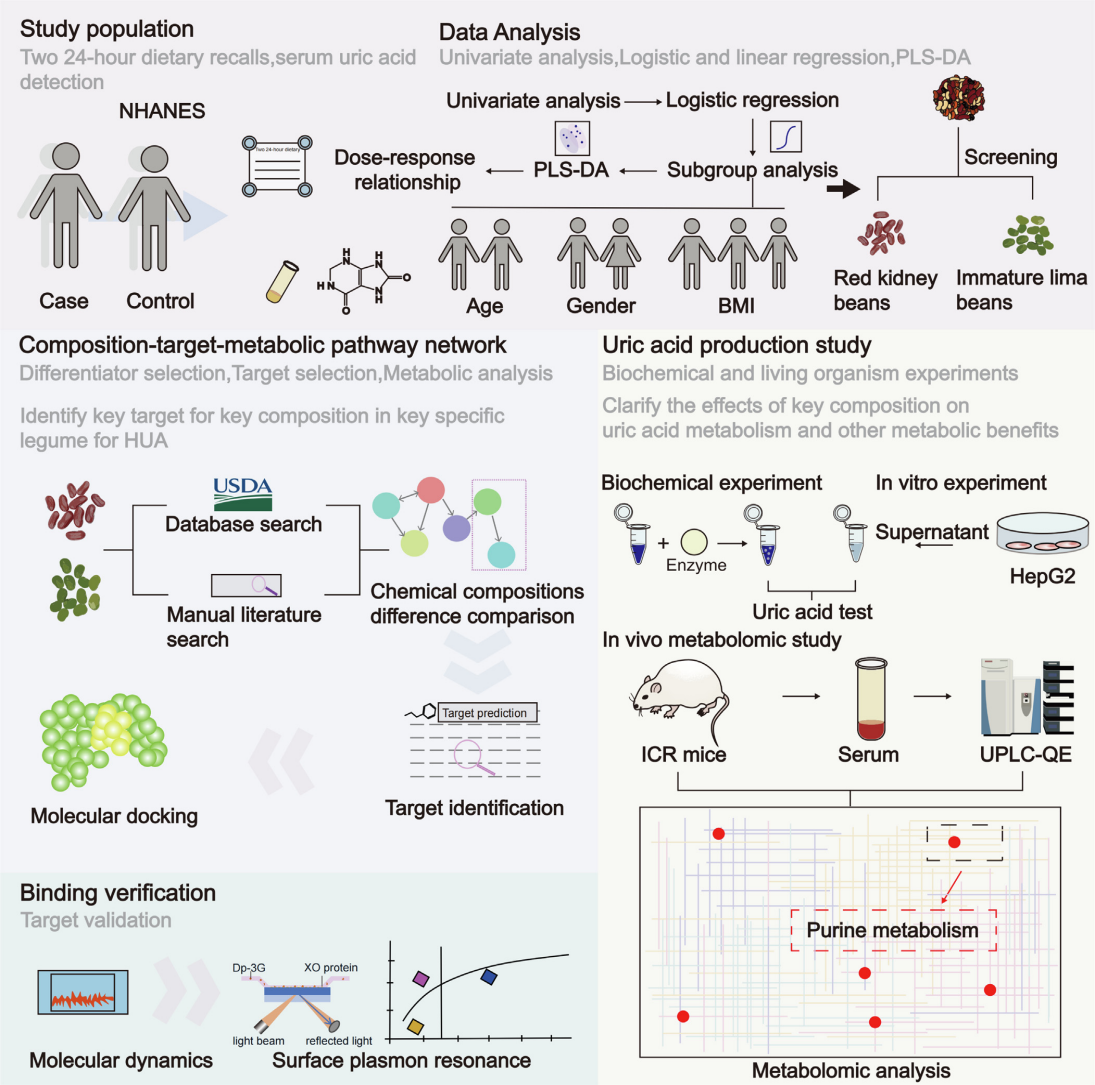
Study design diagram illustrating the four major steps of the study. The first step investigated the association between legume intake and HUA in the representative US population, focusing on specific key legume types. The second step utilized the identified key legume types from the first step and considered the purine metabolic nature of HUA, constructing a novel Composition-Target-Metabolic pathway network to screen key targets and compositions in specific legumes associated with HUA. The third part validated the binding of key proteins and compositions in specific legumes using molecular dynamics simulations and surface plasmon resonance technology. Finally, biochemical and living organism (*in vitro* and *in vivo*) experiments using metabolomic technology were conducted to examine the effects of key compositions in specific legumes on uric acid metabolism and other metabolic benefits.

In this study, we integrated human evidence to explore the relationship between the consumption of 13 specific legume varieties and the prevalence of HUA in a representative US population. Using the principle from the compound-target-pathway (C-T-P) network [12]. we constructed a novel composition-target-metabolic pathway (C-T-M) network (Fig. S1). This network serves as a comprehensive method to analyze specific composition targets and their roles in metabolic pathways affecting HUA. We then employed molecular dynamics (MD) simulations, surface plasmon resonance (SPR), and conducted both *in vitro* and *in vivo* metabolomic studies. These approaches revealed the urate-lowering effect of Dp-3G in red kidney beans by binding to the flavin adenine dinucleotide (FAD) site of the XO enzyme. Our study thus provides novel insights into specific legumes and key natural agents for the prevention and treatment of HUA. The overall experimental design is depicted in Fig. 1.

## Materials and methods

### Ethics statement

All experiments involving animals were conducted according to the ethical policies and procedures approved by the Ethical Review Committee for Experimental Animal Welfare at Nanjing Medical University and strictly adhered to the guidelines outlined by the Institutional Animal Care and Use Committee of Nanjing Medical University (Approval no. IACUC-2312044).

NHANES was approved by the National Center for Health Statistics Research Ethics Review Board, and all participants provided informed consent. Detailed participant information and informed consent procedures are accessible on the official NHANES website (https://www.cdc.gov/nchs/nhanes/index.htm/).

### Human population study

#### Study population

NHANES is a cross-sectional survey of the US population, designed as an ongoing program focusing on various health and nutrition measurements to meet evolving needs. The program is conducted by the National Center for Health Statistics.

This study utilized data from NHANES 2015–2016, encompassing information from participants' 24-hour dietary recall questionnaires, demographic profiles, physical examinations, blood biochemistry analyses, smoking and alcohol consumption surveys, and medical history questionnaires. Exclusion criteria included: (1) Incomplete data from two 24-hour dietary recall sessions; (2) Age less than 18 years or greater than 79 years; (3) Missing information on PIR, education, BMI, serum uric acid levels, smoking and alcohol habits, as well as prevalence of diabetes and hypertension; (4) Recent use (within the past 30 days) of ULT medications such as allopurinol, febuxostat, or probenecid [4]; (5) Consumption of legumes of unspecified varieties.

Initially, 9,971 participants met the inclusion criteria. After excluding 2,944 individuals with incomplete dietary recall data, and further excluding those without serum uric acid data or with unclear legume consumption, 3,957 participants remained. Further exclusions based on age, ULT therapy status, and missing covariate data (BMI, PIR, education, smoking, drinking, hypertension, and diabetes) left a final cohort of 2,676 participants included in this study (Fig. S2).

#### Diagnosis of HUA

Data from the 2015–2016 survey were collected by trained technicians using the Beckman Coulter UniCel DxC 800 Synchron and Beckman Coulter UniCel DxC 660i Synchron analyzers, employing a timed endpoint method to measure participants' serum uric acid levels.

HUA was defined as a serum uric acid level >7.0 mg/dL in men or >6.0 mg/dL in women [13]. The entire study population was categorized into HUA and non-HUA groups based on their serum uric acid levels.

#### Legumes intake measurement and grouping

Based on the food codes from the 2-day, 24-hour dietary review data, in conjunction with the United States Department of Agriculture (USDA) food code database, we screened specific legume intake codes for inclusion in our analysis to prevent exposure misclassification. We cross-referenced the USDA Food and Nutrient Database for the 2015–2016 Dietary Studies (FNDDS) and the Food Code classification criteria through the DRXFCD_I file system. This dual-coding framework includes abbreviated (60 characters) and extended (200 characters) food descriptions, enabling the precise identification of legume components in complex dietary entries, which is according to previous literature [14,15]. Legumes were categorized by type; those with insufficient consumption were excluded from analysis. Peanuts and soybeans were also excluded due to their higher lipid content [8]. Ultimately, the following legume varieties from the NHANES database were included: (1) Black, brown, or Bayo beans (N = 2,012); (2) Chickpeas (N = 1,925); (3) Green peas (N = 1,965); (4) Green string beans (N = 2,165); (5) Immature lima beans (N = 1,914); (6) Lentils (N = 1,934); (7) Peas, cowpeas, field peas, or blackeye peas (N = 1,900); (8) Pinto, calico, or red Mexican beans (N = 2,077); (9) Red kidney beans (N = 1,956); (10) Snowpea (N = 1,902); (11) String beans (N = 1,933); (12) White beans (N = 1,915); (13) Yellow, canary, or Peruvian beans (N = 1,903). Based on consumption, participants were grouped into two categories: those with legume intake and those without. The analysis covered three primary types of dried legumes commonly used in Western cuisine: peas, lentils, and kidney beans.

Additionally, to examine dose–response relationships, USDA food codes from the NHANES two-day dietary recall were harmonized with USDA FPED 2015–2016 codes to quantify legume intake levels. FPED 2015–2016 provides ingredient quantities per 100 g, so individual food intake in grams was divided by 100 and multiplied by the corresponding FPED equivalents. Mean equivalents of legume intake per participant were calculated separately for each of the two recall days [16].

#### Measurement of other indicators and grouping

Gender, age, race, education level, and PIR were obtained from the demographic questionnaire. Race was categorized as non-Hispanic white and other races. Education level was classified into four categories: high school and below, high school graduate/GED or equivalent, some college or AA degree, and college graduate or above.

BMI information was derived from the physical examination data, ensuring consistency with the BMI formula: BMI = weight / height^2^. BMI was grouped into two categories: normal (18.5–24.9 kg/m^2^) and abnormal (<18.5 kg/m^2^ or >24.9 kg/m^2^) [17].

Participants were stratified into two groups: never smoked and smoked, based on their response to the question “Have you smoked at least 100 cigarettes in your entire life?” in the smoking questionnaire [17].

Participants were categorized as drinking or non-drinking based on their response to the question “In any one year, have you had at least 12 drinks of any type of alcoholic beverage?” [17].

Based on blood pressure measurements (hypertension defined as systolic blood pressure ≥130 mmHg and/or diastolic blood pressure ≥80 mmHg), participants were classified as hypertensive or non-hypertensive [18].

According to responses to the diabetes questionnaire question “Other than during pregnancy, have you ever been told by a doctor or health professional that you have diabetes or sugar diabetes,” and considering a glycated hemoglobin level of 6.5 % or higher, participants were categorized as diabetic or non-diabetic [19].

### Construction of C-T-M pathway network

#### Screening of specific compositions of key legumes

In this analysis, we employed the principle of the C-T-P network framework and introduced the novel C-T-M framework to characterize the metabolic features related to HUA (Fig. S1). Following population association research, we identified red kidney beans and immature lima beans as pivotal legumes with distinct associations with HUA. We conducted further investigation into the specific compositions of red kidney beans and immature lima beans using data from the USDA website. We selected the USDA database for target component identification due to its authoritative, standardized nutrient data and global recognition, along with its high compatibility with NHANES coding, ensuring accurate, traceable dietary intake data. This USDA-NHANES integration is widely used in dietary and component studies for its reliability and comprehensive coverage [20,21]. To ensure a standardized comparison, compositions in their raw state were utilized. Through comparative analysis between red kidney beans and immature lima beans, we pinpointed key differential compounds specific to red kidney beans. Additionally, we employed manual screening via Google Scholar as a supplementary method to identify specific compositions of red kidney beans. This approach focused on differentiating compositions reported in current literature between red kidney beans and immature lima beans. We reviewed studies published between January 2003 and December 2023. The searches were conducted independently by two reviewers, with discrepancies resolved through consensus or consultation with a third reviewer as needed.

#### Accessing potential targets of specific compositions and HUA

Detailed information about specific compositions can be found on PubChem (https://pubchem.ncbi.nlm.nih.gov/), a valuable database for accessing biological activity information on small organic molecules. Supported by the National Institutes of Health and maintained by the National Biotechnology Information Center [22], PubChem provides access to chemical structures of compounds. PharmMapper (https://www.lilab-ecust.cn/pharmmapper/) is a platform supported by TargetBank, DrugBank, Binding DB, and PDTD databases. It allows for the identification of potential targets of bioactive small molecules. Similarly, SwissTargetPrediction is a web-based tool used for predicting targets of such molecules. To identify targets of specific compositions, we imported chemical structures or SMILES obtained from PubChem into PharmMapper and SwissTargetPrediction. In predicting potential targets, given the common established practices in the literature, we used PharmMapper with screening criteria of a z-score >0.9 and a Norm Fit >0.6 [23,24], and selected the top 15 predictions with probability >0.1 from SwissTargetPrediction [[25], [26], [27]]. For HUA target identification, we applied selection criteria of Relevance Score >10 in GeneCards and Inference Score >50 in CTD to obtain HUA targets [28,29]. Subsequently, these targets were translated into gene IDs and gene symbols.

#### Metabolic network analysis for targets of specific compositions and HUA

MetaboAnalyst 6.0 (https://genap.metaboanalyst.ca/) was utilized for KEGG Global Metabolic Network analysis to identify shared targets, conduct pathway enrichment, and construct networks. Given that HUA is associated with abnormal uric acid metabolism in the purine metabolism pathway, our focus was on the intersection of identified potential targets with genes in this pathway. Key genes identified from PharmMapper and SwissTargetPrediction were inputted into the KEGG website (https://www.genome.jp/kegg/) for pathway visualization and metabolic network construction, aiming to elucidate the underlying metabolic mechanisms. As a supplement, we searched for other HUA-related metabolism by KEGG enrichment of genes from GeneCards and CTD.

### Molecular docking

#### Protein and small molecule structure preparation

Initially, the three-dimensional structure file of the protein was obtained from the RCSB PDB database (https://www.rcsb.org/). Following the download, an extensive pre-processing of the protein structure was conducted. This involved procedures such as hydrogenation, computation of atomic charges, and energy minimization to optimize the protein structure. Simultaneously, small molecule structure files were sourced from PubChem. These small molecules underwent structure optimization and charge assignment to ensure structural accuracy and stability in molecular simulations.

#### Molecular docking and result analysis

After completing the preprocessing of the protein structure, the processed PDB file was converted into suitable input files for molecular docking using the LePro module of LeDock [30]. This step ensured full compatibility between the protein structure and the LeDock software, thereby ensuring the accuracy of the molecular docking process. Subsequently, molecular docking was performed using LeDock, and the optimal docking structure was selected. This structure, along with the protein, formed the protein–ligand complex. The complex structure was then imported into Maestro 13.5 software. Here, the interactions between the ligand and the receptor were analyzed using the Interactions Toggle and 2D Sketcher modules.

### MD simulations

#### Construction of MD simulation system

The molecular docking results were ranked based on their scoring, and the lowest scoring complex was selected for further construction of the MD system. Initially, acpype was employed to compute the charges of small molecules and generate a topology file. To set up the solvation system, the protein–ligand complex was centered, and the editconf module within Gromacs 2023 software was utilized to define a cubic box dimension [31]. Subsequently, the genbox command was used to insert TIP3P water molecules into the box, creating a suitable solvation environment around the complex [32]. Appropriate amounts of Na^+^ and Cl^-^ ions were added randomly to the solvent box using the grompp and genion commands, respectively [31]. Finally, a solvated system under periodic boundary conditions with an appropriate size and electroneutrality was established, preparing the system for subsequent MD simulations.

#### MD simulation-energy minimization

To alleviate forces stemming from unreasonable atomic coordinates in the initial conformation, energy minimization of each solvated protein–ligand complex system was conducted prior to formal MD simulations. The steepest descent method was employed with an energy threshold of 1,000 kJ·mol^−1^·nm^−1^, allowing up to 50,000 steps of energy optimization to attain a reasonable level of system energy and alleviate atomic stress.

#### Equilibration in the isothermal-isotropic (NVT) ensemble

Following energy minimization, equilibrium simulations of the complex were conducted under the NVT ensemble to gradually raise the system temperature and achieve equilibrium. The control parameters were set as follows: the leap-frog integrator was employed for integration, with a simulation step size of 2 fs, and a total simulation duration of 100 ps (50,000 steps). Temperature coupling utilized the V-rescale method [33] with a reference temperature of 300 K and a coupling constant of 0.1 ps. Separate temperature coupling was applied to both the protein–ligand complex and the solvent.

During the simulation, all bond lengths were constrained using the LINCS algorithm [34], while bond lengths involving hydrogen atoms remained unchanged. The parameters for electrostatic and van der Waals interactions were consistent with those used during the energy minimization stage. To restrict conformational changes of the protein, a position restraint of 1,000 kJ·mol^−1^·nm^−2^ was applied to the heavy atoms of the protein. Energy data and conformations were saved every 500 steps during the simulation.

#### Equilibration in the isothermal and isobaric (NPT) ensemble

Following NVT equilibrium, a 100 ps equilibrium simulation of the complex was conducted under the NPT ensemble to achieve equilibrium in system density and pressure. The pressure coupling utilized the Parrinello-Rahman method [35], with a reference pressure set at 1.0 bar, a pressure coupling constant of 2.0 ps, and an isothermal compressibility coefficient of 4.5 × 10^−5^ bar^−1^. Temperature control parameters remained consistent with those used in the NVT equilibrium stage. Positional constraints were applied to the protein during the simulation to maintain its conformational stability. All other parameter settings were kept identical to those used in the NVT equilibrium stage. Energy data and conformation snapshots were saved every 500 steps for subsequent analysis.

#### Production stage simulation

Following energy minimization and pre-equilibration of both ensembles, the protein–ligand complex system achieved a more stable initial state. Building upon this foundation, we conducted MD simulations over 200 ns to capture dynamic changes within the complex. Integration was performed using the leap-frog method with a simulation step size of 2 fs, totaling 25,000,000 steps. Throughout the simulation, temperature and pressure coupling parameters remained consistent with those used in the NPT equilibrium stage, maintaining the system at 300 K and 1 atmosphere. The cutoff scheme and handling methods for van der Waals and electrostatic interactions were kept consistent with previous stages. For ease of subsequent analysis, energy information was recorded every 5,000 steps (10 ps), and trajectory files were compressed at the same interval.

#### SPR binding analysis

The small molecule Dp-3G (Chengdu Caoyuankang Bio-Technology Co., Ltd., purity >98 %) was diluted to 0.78 μM using running buffer (1 × PBS-P^+^) for a 2-fold serial dilution. For proteins, the XO sample (Sigma-Aldrich, X4376) was diluted to 40 μg/mL using sodium acetate at pH 5.0. SPR analysis was conducted using a Biacore T200 instrument equipped with a CM5 sensor chip to study protein-molecule interactions. On the experimental channel Fc2, the chip was activated with an EDC and NHS mixture (1:1) for 420 s, resulting in fixation at approximately 13,600 resonance units (RU), followed by ethanolamine (EA) flowing through the chip surface to block excess sites [36]. The reference channel Fc was activated and directly blocked. Analytes flowed through both experimental and reference channels with a binding time of 60 s, a flow rate of 50 μL/min, and a dissociation time of 60 s. The regeneration buffer used was 1 × PBS-P^+^, with a regeneration time of 30 s and a flow rate of 30 μL/min. Using the T200′s analysis software (version 3.2), we selected appropriate continuous concentrations for Kinetic 1:1 Binding or Steady State Analysis. The affinity between proteins and small molecules is determined by their dissociation constants (KD values), typically within the concentration range of 10^-3^ to 10^-9^ M. These values indicate the strength of binding between the protein and the small molecule: a KD value of 10^−3^ M suggests relatively weak binding, approximately 10^−6^ M indicates moderate binding, and around 10^−9^ M indicates strong binding [37]. Most antibodies exhibit KD values in the low micromolar (10^−6^) to nanomolar (10^−7^ to 10^−9^) range.

#### XO reaction-UHPLC/MS coupling analysis

As per literature sources [38], the uric acid production, indicative of Dp-3G's XOI activity, was determined using the XO reaction-UHPLC coupling method. Ultrapure water served as the negative control, while AP (100 μM) acted as the positive control. Test solutions containing Dp-3G at the desired concentration (100 μM) were freshly prepared immediately prior to experimentation. In brief, 50 μL of the test sample, negative control, or positive control solution was mixed with 75 μL of xanthine (0.84 mM) and incubated at 25 °C for 5 min. The reaction commenced upon addition of 100 μL of XO (0.025 U/mL, Sigma-Aldrich, X4376), followed by incubation for 10 min at 25 °C. The reaction was terminated by adding 80 μL of 1 M HCl, yielding the test sample, negative control, or positive control reaction mixtures. The production of uric acid in each group was analyzed using the UHPLC-Q-Exactive platform (UHPLC Ultimate 3000 system, Dionex, Germering, GER; Q-Exactive Orbitrap, Thermo Fisher Scientific, Bremen, GER). To further investigate the concentration-dependent inhibitory effects of Dp-3G, we additionally tested Dp-3G at concentrations of 0, 100, and 200 μM. The experimental procedure remained consistent with the method described above. We employed randomization, concealed allocation, and blinded data analysis throughout the following experiments to ensure that the study was conducted in a blinded manner.

### In vitro and *in vivo* metabolomic experiments

#### Cell experiment

HepG2 human-derived hepatocellular carcinoma cells were procured from Wuhan Punosai Life Science and Technology Co. These cells were cultured in DMEM medium supplemented with 10 % FBS, 1 % streptomycin (0.17 mM), and 1 % penicillin (0.3 mM). The cells were maintained in a humidified atmosphere with 5 % CO_2_ at 37 °C.

Following the methodology outlined in reference [39], HepG2 cells were grown in DMEM complete medium until they reached 85–95 % confluence. Subsequently, they were seeded into 24-well plates at a density of 1 × 10^5^ cells/mL. The cells were then incubated at 37 °C for 18 h to ensure adherence. Phosphate-buffered saline (PBS, 0.01 M) was used to wash the cells. Next, the culture medium was replaced with fresh medium containing 1.25 mM adenosine and incubated for an additional 24 h. Afterward, the medium was aspirated, and the cells were washed with PBS 2–3 times. For the experimental treatments, the cells were cultured in complete medium containing 0.025 U/mL XO (Sigma-Aldrich, X4376) with or without Dp-3G (100 μM) or AP (100 μM) for 24 h. Finally, the uric acid content in the supernatant was analyzed using the UHPLC-Q-Exactive platform (UHPLC Ultimate 3000 system, Dionex, Germering, GER; Q-Exactive Orbitrap, Thermo Fisher Scientific, Bremen, GER) for each experimental group.

### In vivo metabolomic experiments

#### Animals and treatment

Eight-week-old male ICR mice, meeting SPF-grade criteria, were procured from the Laboratory Animal Center at Nanjing Medical University (Nanjing, China). The mice had ad libitum access to food and water and were housed in a controlled environment under a 12-hour light/dark cycle at temperatures ranging from 20 to 26 °C.

The mice were randomly assigned to three groups: (1) potassium oxonate (PO) group (8 mice per group); (2) PO + AP group (6 mice per group); (3) PO + Dp-3G group (3 mice per group). The number of animals used in our study was determined using the “power analysis” method described in 3Rs-Reduction.co.uk, ensuring adequate statistical power (α = 0.05, two-sided) based on our pilot study. The sample size was also according to previous similar studies [40,41]. The HUA model was established according to a standard protocol using the uricase inhibitor PO, and the experiments examining the treatment's effect on serum uric acid were conducted following previous studies that utilized this HUA model [42,43]. One hour prior to treatment, mice were intraperitoneally injected with PO (200 mg/kg) to elevate serum uric acid levels. Given the fact that Dp-3G showed a predominance of parent compounds in long-term oral intake experiments[[44], [45], [46]], for comparative purposes, Dp-3G (100 mg/kg) was administered intraperitoneally one hour after PO administration, while AP (5 mg/kg) served as the reference control. The intraperitoneal dosages were selected based on previous reports [43,47]. Additionally, oral gavage experiments of DP-3G and AP were conducted, where Dp-3G was administered orally at 400 mg/kg (4 mice per group) one hour after oral PO administration, with AP (10 mg/kg) (5 mice per group) as a positive control. The oral dosages were selected based on previous reports [48,49]. The following studies were performed in a blinded fashion. The mice were randomly divided into control group, AP treated group and Dp-3G treated group, and the grouping information was sealed by an independent person. AP and Dp-3G drug solutions were prepared by a third party to ensure consistent appearance, volume, and administration mode. Dosing procedures and samples were performed by investigators who were unaware of the group assignments. The data analysis was blinded during the whole process [50].

#### Organ coefficient

The mice were weighed and euthanized. Tissues, including the liver and kidney, were then dissected and weighed. Subsequently, the organ coefficients of the liver and kidney relative to body weight were calculated as the ratio of tissue weight (wet weight, g) to body weight (g).

#### Pathological histology

For histopathological examination, kidney and liver tissues were fixed in 4 % paraformaldehyde and subsequently stained with H&E for observation.

#### Xanthine oxidase (XO) activity Assay

The activity of XO in the liver was measured using the Xanthine Oxidase Activity Assay Kit (Solarbio, BC1095) in accordance with the manufacturer's protocol.

#### Metabolomic analysis

Serum metabolomic analysis was conducted using the UHPLC-Q-Exactive platform (UHPLC Ultimate 3,000 system, Dionex, Germering, GER; Q-Exactive Orbitrap, Thermo Fisher Scientific, Bremen, GER) following previous protocol [51,52]. Initially, protein precipitation was performed using a methanol–water mixture, and the resulting supernatant was concentrated to dryness using a Labconco centrifugal concentrator (Kansas, MO, USA). The dried samples were reconstituted and prepared for analysis. All sample injections were randomized to prevent injection order bias. Chromatographic separation was carried out using a Hypersil GOLD C18 column (100 mm × 2.1 mm, 1.9 μm, Thermo Fisher Scientific, Vilnius, Lithuania) with a column temperature set at 40 °C and a flow rate of 0.40 mL/min. Mobile phase A consisted of acetonitrile containing 0.1 % formic acid, while mobile phase B was ultrapure water containing 0.1 % formic acid. Mass spectrometry utilized heated electrospray ionization (HESI) with a spray voltage of 3.5 kV for positive ion mode and 2.5 kV for negative ion mode. Full scan mode was employed with a scan range of 70–1,050 *m*/*z* and a resolution of 70,000. Metabolite identification involved comparing accurate mass and retention time with those of authentic metabolite standards using a self-built standard library in TraceFinder 5.1 software (Thermo Fisher Scientific). Statistical modeling was performed using the OPLS-DA model in SIMCA-P (Version 14.1, Umetrics). Pathway analyses were conducted using the pathway analysis and enrichment analysis modules in MetaboAnalyst 6.0. Additionally, a pathway-based network of metabolomic data was established using the MetScape plugin (Version 3.1.3) for Cytoscape (Version 3.10.2). No unexpected or unusually high safety hazards were encountered during any of the experiments. In XO reaction-UHPLC/MS coupling analysis and metabolomic experiments, the within-run sequence of a sample is random (established by random number generation) [53], to avoid complications related to the injection order. This is also consistent with previously published methods [51,54].

#### Literature search for HUA-related diseases: Implications of identified metabolites

We searched for studies published before July 2024 from the databases of PubMed and Google Scholar. The search terms included a list of standard names of key metabolites in our study, HUA-related diseases, such as atherosclerosis, hypertension, chronic kidney disease, arthritis coupled with *in vitro* cellular models commonly used for the corresponding diseases, such as vascular smooth muscle cells and vascular endothelial cells for atherosclerosis and hypertension, and renal tubular epithelial cells for chronic kidney disease. Manual searches were also performed based on database results. The objective of this literature search was to provide current insights into the associations between relevant metabolites and HUA-related diseases across four layers of evidence: human studies, animal studies, *in vitro* cellular studies, and mechanistic studies. Inclusion criteria for literature in our study were as follows: A) publications that reported statistically significant associations between metabolites and HUA-related diseases or significant effects of metabolites on these diseases; B) the reported associations or effects and their directions were supported by other independent biological evidence across the four layers to avoid including controversial conclusions contrary to consensus and potential publication bias. Study selection, data extraction, and validity assessment involved three reviewers to minimize bias and error. Disagreements were resolved through discussion, and consensus was achieved among all three reviewers.

### Statistical analysis

For categorical variables, frequencies and percentages described differences between the HUA and non-HUA groups, with Fisher’s exact test used to test these differences. For continuous variables, differences were compared using independent t-tests. When comparing among three groups, one-way analysis of variance (ANOVA) followed by Dunnett's test was applied. In the HUA mouse model, the dose–response relationship between Dp-3G treatment and uric acid levels was evaluated using Pearson correlation analysis.

Differences in legume intake between HUA and non-HUA groups were initially analyzed with Fisher’s exact test. Changes in continuous legume intake were compared between these groups using t-tests. The association between specific legume intake and HUA risk was assessed using logistic regression, where legume intake status (independent variable) and HUA prevalence (dependent variable) were considered. OR and their 95 % CI were calculated for each group. Three models were constructed: Model 1 (unadjusted), Model 2 (adjusted for age, gender, race, education level, PIR, BMI, smoking, and alcohol use), and Model 3 (fully adjusted, including hypertension and diabetes in addition to Model 2 variables).

Subgroup analyses were conducted based on age, BMI, and gender. Age subgroups were divided into <55 years and ≥55 years [55]. BMI was categorized as normal and abnormal, with the abnormal group further divided into overweight (24.9 < BMI ≤ 29.9 kg/m^2^) and obesity (BMI > 29.9 kg/m^2^) [17]. Due to the small number of individuals with low BMI, this category was not included in the analysis. The gender subgroup consisted of males and females. In each subgroup, corresponding covariates were excluded from the logistic regression analysis.

To further investigate the association between specific legume intake and the risk of HUA under mixed legume exposure, we conducted Partial Least Squares Discriminant Analysis (PLS-DA) using the Mixomics package (version 6; R Development Core Team) in R (version 4.0.4) software. PLS-DA has become increasingly significant due to its versatile applications, which not only reduces data dimensionality but also facilitates feature selection and categorization of information [56]. To consider the effect of weights on the results between HUA and key legume intake, we performed sensitivity analyses with weights as covariates to capture relevant factors, such as where individuals live, their demographic characteristics [57].

Then, we analyzed the dose–response relationship by constructing logistic regression models for HUA and intake levels of red kidney beans and immature lima beans using the fully adjusted model set. The intake levels of red kidney beans and immature lima beans were logarithmically transformed. To prevent issues arising from zero intakes during logarithmic calculations, a small constant (0.001) was added to the data before transformation [58]. To explore the association between intake levels of red kidney beans and immature lima beans with serum uric acid levels, uric acid levels were categorized into three groups (normal, moderately elevated, severely elevated) based on gender-specific thresholds. For men, these groups were defined as (1) 3.4–7.0 mg/dl, (2) 7.1–8.9 mg/dl, and (3) ≥9.0 mg/dl; while for women, the categories were (1) 2.4–6.0 mg/dl, (2) 6.1–7.9 mg/dl, and (3) ≥8.0 mg/dl according to established standards [59,60]. Intake levels of legumes were categorized into three groups: for red kidney beans, these were (1) no intake, (2) 0–3.75 oz. equivalents (oz. eq.), and (3) >3.75 oz. eq.; whereas for immature lima beans, the categories were (1) no intake, (2) 0–0.35 cup equivalents (cup eq.), and (3) >0.35 cup eq [61]. We used linear regression using the full adjusted model set.

Statistical assumptions were tested at a significance level of α = 0.05. To address multiple comparisons, we employed a combined statistical significance criterion of a two-sided test with *P* < 0.05 and VIP > 1 in PLS-DA for the analysis of legume and metabolomic data [62]. All analyses were conducted using R software (version 4.0.4). The workflow of the study is illustrated in Fig. S3. The study consisted of four key stages: a human population study, the construction of C-T-M pathway network, validation through binding assays, and an investigation of uric acid production using biochemical and *in vivo* experiments, as depicted in Fig. S3.

## Results

### Intake of specific legume species and HUA in humans

#### The study included 2,676 participants from the US representative population

Following the inclusion and exclusion process (Fig. S2), this study comprised 2,676 eligible subjects, including 466 in the HUA group and 2,210 in the non-HUA group. The basic characteristics of the study subjects in both groups are detailed in Table S1. Compared to the non-HUA group, the HUA group was characterized by older age, a higher proportion of males, a greater percentage of non-Hispanic White individuals, a higher prevalence of abnormal BMI, increased incidence of hypertension and diabetes (22.7 % vs. 14.8 %), and elevated uric acid levels (7.53 vs. 4.96 mg/dl).

#### Univariate and multivariate models identified that the consumption of red kidney beans reduced the risk of HUA

**Fig. 2:**
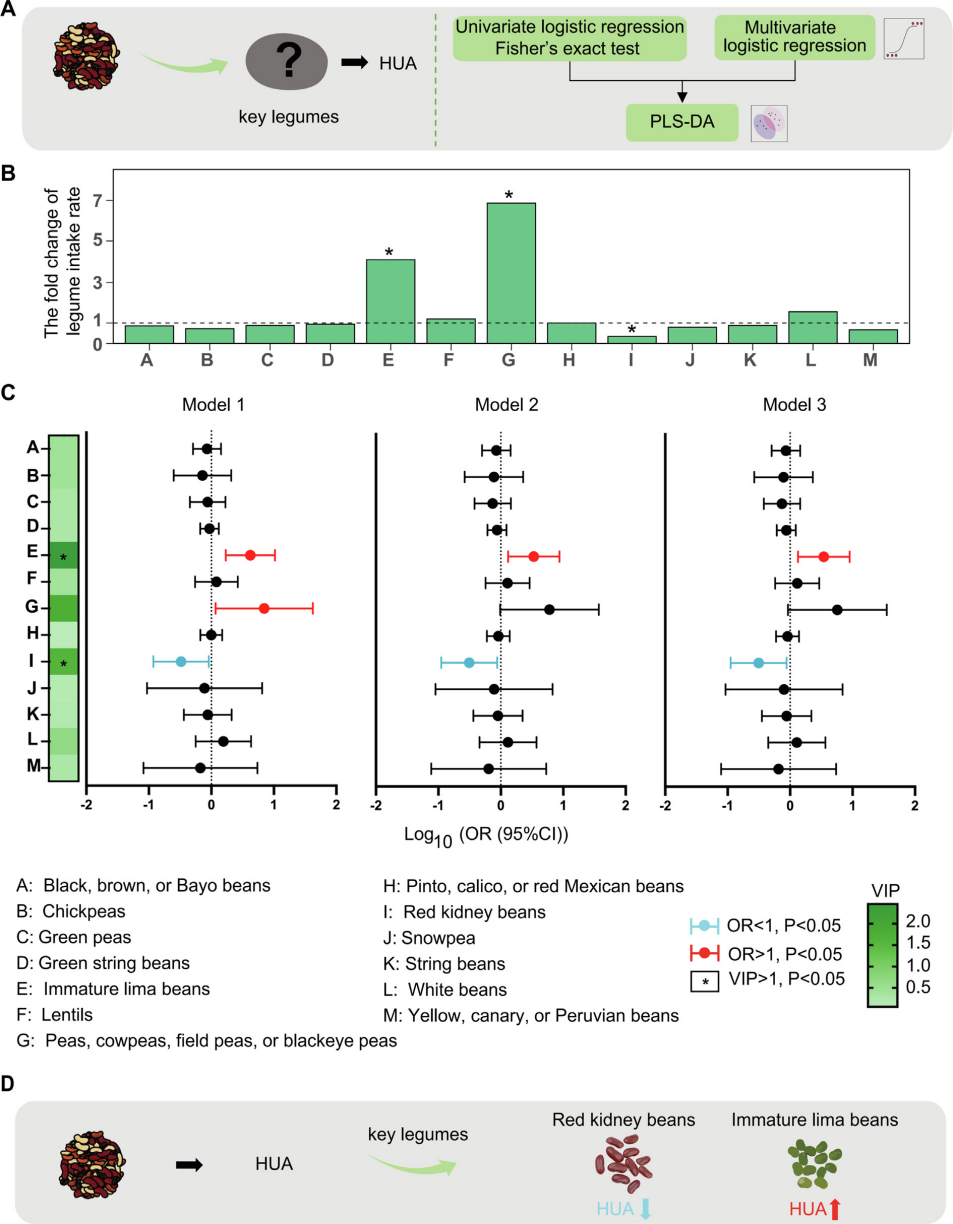
Results of the association between legume intake and HUA in the representative US population. (A) Study design aimed at identifying key legume intakes influencing HUA. (B) Bar charts summarizing the fold change in legume intake rates between the HUA and non-HUA groups, highlighting significant changes including peas, cowpeas, field peas (blackeye peas), immature lima beans, and red kidney beans. (C) OR and 95 % CI for the association between legume intake and HUA risk in Models 1–3, as well as VIP scores from PLS-DA, identifying robust associations particularly with immature lima beans and red kidney beans. (D) Robust and significant associations between intake of immature lima beans and red kidney beans with HUA, considering mixed legume intake scenarios. Abbreviations: OR: Odds ratios; CI: confidence intervals; VIP: Variable Importance in Projection; PLS-DA: Partial Least Squares Discriminant Analysis. (For interpretation of the references to color in this figure legend, the reader is referred to the web version of this article.)

Various analyses were conducted to examine the associations between legume intake and HUA (Fig. 2A). Fisher's exact test (Table S2 and Fig. 2B) revealed significant differences in the intake rates of red kidney beans, immature lima beans, and peas, cowpeas, field peas, or black-eyed peas between the two groups (*P* < 0.05). Interestingly, the intake rate of red kidney beans was significantly higher in the non-HUA group than in the HUA group (*P* = 0.024), suggesting a potential protective effect against HUA. The logistic regression results of Model 1, Model 2, and Model 3 are presented in Table S3. In Model 1, the intake of immature lima beans significantly increased the risk of HUA (OR = 4.21, 95 % CI 1.70–10.43), while the intake of peas, cowpeas, field peas, or blackeye peas also significantly raised the risk (OR = 7.01, 95 % CI 1.17–42.12). Importantly, consuming red kidney beans significantly decreased the risk of HUA (OR = 0.33, 95 % CI 0.12–0.91) (Fig. 2C), affirming the potential effects of these legume varieties on HUA risk. To further adjust for confounding factors, partially adjusted Model 2 and fully adjusted Model 3 were constructed. Importantly, the significant associations of intake of immature lima beans and red kidney beans with HUA risk persisted with slight changes in ORs (Fig. 2C), underscoring the robustness of the relationships between intake of immature lima beans, intake of red kidney beans, and HUA risk.

Based on statistical and biological considerations, subgroup analyses were conducted considering age, BMI, and gender (Fig. S4A). Among those aged <55 years, consuming red kidney beans was associated with reduced HUA risk, while among those aged ≥55 years, intake of immature lima beans was linked to elevated HUA risk (Table S4 and Fig. S4B, C). Additionally, a significant association was observed between intake of red kidney beans and immature lima beans and HUA risk in the abnormal BMI and obesity groups (Table S5 and Fig. S4D-G). No significant correlations were found between various legume consumptions and HUA risk by gender (Table S6 and Fig. S5).

#### Mixed exposure and sensitivity analysis confirmed the above association

It was observed that within the total population, consumption of three specific groups of legumes (immature lima beans; peas, cowpeas, field peas, or blackeye peas; red kidney beans) showed VIP values above 1.00 in the PLS-DA model for mixed exposure effects (Fig. 2C and Table S7), indicating a robust and significant association between consumption of immature lima beans and red kidney beans with HUA risk under mixed legume intake (Fig. 2D).

Furthermore, VIP results confirmed the robustness of the association between intake of immature lima beans in individuals aged 55 years or older, and intake of red kidney beans in those under 55, with HUA. Additionally, it highlighted the significant association between intake of immature lima beans and red kidney beans in obese individuals with HUA (Table S7 and Fig. S4B-H).

Moreover, the study demonstrated that in cases of highly variable sample weights or when covariates used for weighting (e.g., age, gender, race) are already included in the regression model, unweighted estimation is recommended [63]. However, to assess the impact of survey weights on outcomes, survey weights were added to the regression models, confirming that the associations between intake of immature lima beans and red kidney beans with HUA remained robust (Table S8).

#### A dose–response relationship was found between the consumption of red kidney beans and serum uric acid levels

Through t-tests, we found significant differences in the intake levels of red kidney beans (*P* = 0.005) and immature lima beans (*P* = 0.025) between the HUA and non-HUA groups (Table S2). We observed that the risk of HUA was negatively correlated with red kidney beans intake levels in a dose-dependent manner (*P* = 0.035, OR = 0.71, 95 % CI 0.52–0.98), while it was positively correlated with immature lima beans intake levels (*P* = 0.009, OR = 1.65, 95 % CI 1.13–2.41) (Table S9 and Fig. S6A, B). Further analysis revealed significant associations between intake levels of red kidney beans and immature lima beans and serum uric acid levels (*P* = 0.024, β = -0.12, 95 % CI −0.22 to −0.02 for red kidney beans; *P* = 0.007, β = 0.18, 95 % CI 0.05–0.31 for immature lima beans). These findings suggest that increased intake of immature lima beans and red kidney beans may alter the risk of HUA by affecting serum uric acid levels.

### Construction of C-T-M pathway network for key legumes reveals underlying mechanisms

#### Anthocyanins and folate: Key differences between red kidney beans and immature lima beans

Due to the complex compositions in legumes, their overall effects result from a balance of urate-lowering and increasing components [64]. Therefore, the human population study indicates a high abundance of urate-lowering components in red kidney beans, whereas immature lima beans lack them. Chemical compositions of immature lima beans and red kidney beans were obtained through the USDA database (Fig. S7A). 5-Methyltetrahydrofolate (5-MTHF), the highly bioactive form of folate and a type of B-vitamin commonly referred to as folate [65], was identified as the key difference between red kidney beans and immature lima beans, selected for further analysis. We examined the Australian Food Composition Database (https://www.foodstandards.gov.au/science-data/monitoringnutrients/afcd), another source that consistently identified notably high folate levels in red kidney beans, further reinforcing the validity of our conclusions. The relevant results are presented in Fig. S8.

Concurrently, a literature review was conducted to explore differences in chemical compositions, identifying anthocyanins as key differential compounds. Studies show that the components of red kidney beans differ significantly from those of other legumes, such as lima beans, with anthocyanin type and content standing out the most [66]. Anthocyanins are primarily present in the colored seed coats of leguminous plants, explaining the color disparity between the two legume types [67]. Immature lima beans, harvested before full maturity, exhibit a typical green color, while red kidney beans have a red seed coat. To pinpoint specific anthocyanins in red kidney beans, a manual literature search was performed (Fig. S7B). Using Google Scholar, we searched “red kidney beans” and “anthocyanins” as keywords, retrieving 506 articles. After reviewing titles and abstracts, 415 irrelevant papers were excluded, leaving 91 papers. Of these, 27 were removed due to unavailable full texts. The remaining 64 full texts were evaluated, and 61 were excluded for not specifying anthocyanin concentrations or content, resulting in 3 included articles. These identified cyanidin-3-glucoside (Cy-3G) and Dp-3G as key anthocyanins in red kidney beans, providing evidence of higher levels of cyanidin-3-glucoside (Cy-3G) and Dp-3G in red kidney beans [66,68,69]. In summary, the key differential compounds between red kidney beans and immature lima beans were identified as folate (5-MTHF) and anthocyanins (Cy-3G and Dp-3G).

#### Potential targets for anthocyanins and folate: IMPDH2, PDE4D, and XO

**Fig. 3:**
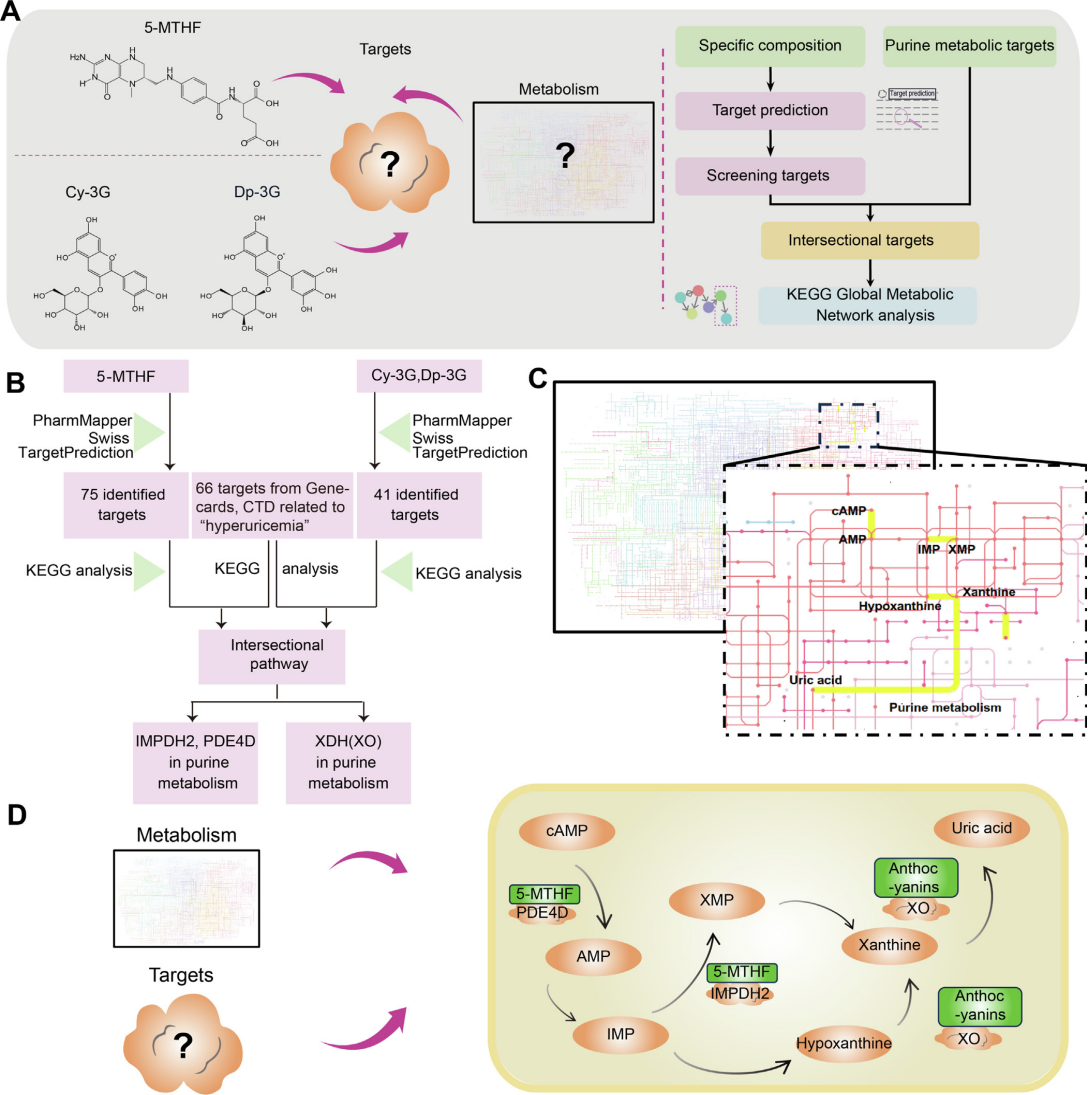
Results of the identification of key targets for key compositions (Cy-3G, Dp-3G, and folate) in red kidney beans. (A) Research design aimed at identifying metabolic targets related to HUA for folate (5-MTHF) and anthocyanins (Cy-3G and Dp-3G). (B) Flow chart illustrating target identification followed by intersection with the KEGG HUA-related pathway. IMPDH2 and PDE4D for 5-MTHF, and XO for Cy-3G and Dp-3G, were identified as key targets in purine metabolism. (C) KEGG Global Metabolic Network analysis used to screen for intersecting targets, perform metabolic pathway enrichment, and construct a metabolic network. (D) Utilization of the KEGG database to display the proteins targeted by key compositions (IMPDH2, PDE4D for 5-MTHF, XO for Cy-3G and Dp-3G) within the purine metabolism pathway relevant to HUA. Abbreviations: 5-MTHF: 5-Methyltetrahydrofolate; Cy-3G: cyanidin-3-glucoside; Dp-3G: delphinidin-3-glucoside; IMPDH2: Inosine-5′-monophosphate dehydrogenase 2; PDE4D: Phosphodiesterase 4D; XO: Xanthine oxidase. (For interpretation of the references to color in this figure legend, the reader is referred to the web version of this article.)

We aimed to identify potential targets for 5-MTHF and anthocyanins (Cy-3G and Dp-3G) (Fig. 3A). The structures' sdf files and smiles were processed through PharmMapper and SwissTargetPrediction (Fig. 3B). We next conducted KEGG Global Metabolic Network analysis using Metaboanalyst 6.0 (https://genap.metaboanalyst.ca/) to identify intersectional targets. Given that HUA results from abnormal uric acid levels involving the purine metabolism pathway, our focus was on the overlap between identified potential targets and genes within this pathway. In addition, we obtained 66 potential HUA targets through GeneCards and CTD. KEGG enrichment analysis of these HUA targets broadened the metabolic pathways associated with HUA, with the purine metabolic pathway also being prominently featured among the enriched pathways of HUA targets. Our analysis highlighted three genes: Inosine-5′-monophosphate dehydrogenase 2 (IMPDH2) and Phosphodiesterase 4D (PDE4D) in relation to 5-MTHF, and XDH (XO) concerning anthocyanins (Cy-3G and Dp-3G). These genes were notably enriched within the purine metabolism pathway (Fig. 3C, D and Fig. S9). Other KEGG enrichment pathways for HUA targets are shown in Tables S1–S11.

#### Molecular docking reveals potential mechanisms of 5-MTHF and anthocyanins (Dp-3G and Cy-3G) in reducing HUA risk

**Fig. 4:**
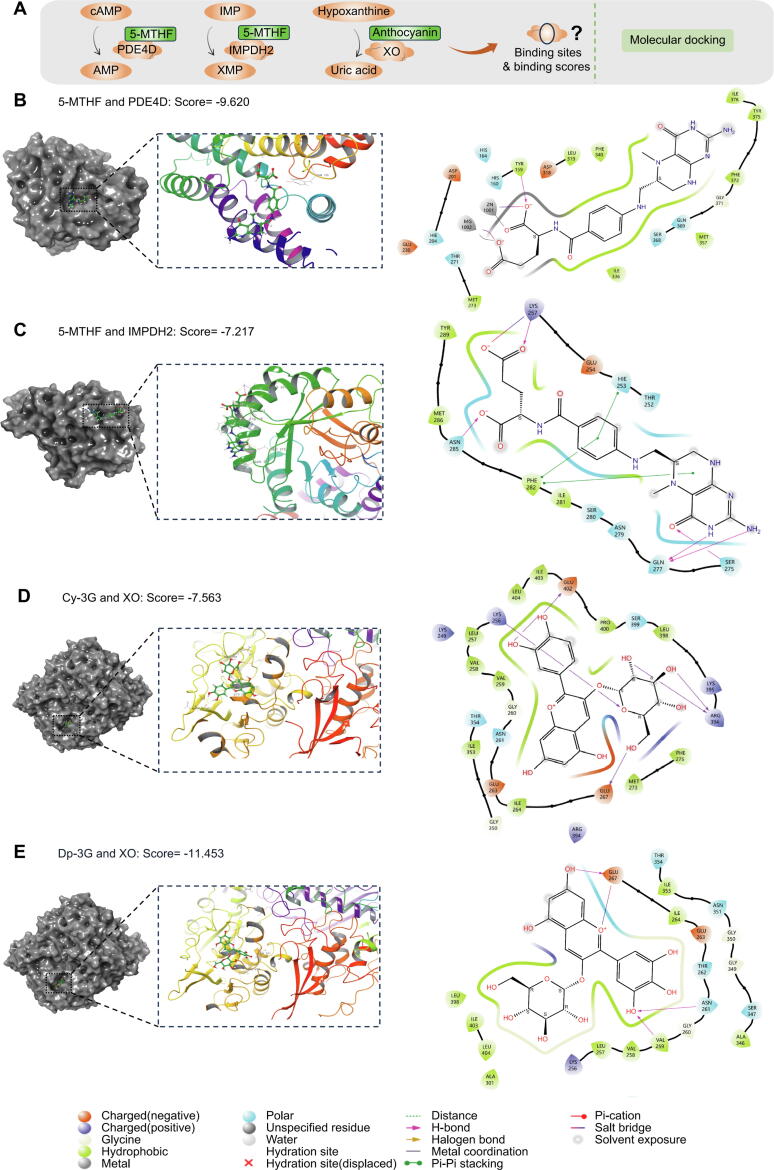
Results of molecular docking analysis. (A) Design for molecular docking of key chemical compositions (Cy-3G, Dp-3G, and 5-MTHF) with key targets (IMPDH2, PDE4D for 5-MTHF, and XO for Cy-3G and Dp-3G). (B) Molecular docking analysis of 5-MTHF and PDE4D. (C) Molecular docking analysis of 5-MTHF and IMPDH2. (D) Molecular docking analysis of Cy-3G and XO. (E) Molecular docking analysis of Dp-3G and XO. In the above dockings, the key chemical compositions effectively bind to their respective targets. Abbreviations: 5-MTHF: 5-Methyltetrahydrofolate; Cy-3G: cyanidin-3-glucoside; Dp-3G: delphinidin-3-glucoside; IMPDH2: Inosine-5′-monophosphate dehydrogenase 2; PDE4D: Phosphodiesterase 4D; XO: Xanthine oxidase.

To gain deeper insights into the potential mechanisms of legumes in managing HUA, we selected key genes and conducted molecular docking to assess their binding capabilities (Fig. 4A). We began by focusing on 5-MTHF, docking it separately with PDE4D (PDB ID: 1xom) and IMPDH2 (PDB ID: 1b3o) (Fig. 4B, C). Simultaneously, we assessed the interaction of the key enzyme XO (PDB ID: 2ckj) with Cy-3G and Dp-3G [70,71]. Cy-3G exhibited a docking score of −7.563 with XO. Dp-3G also showed binding affinity with XO, forming hydrogen bonds with XO's GLU267, ASN261, VAL259, and engaging in a salt bridge with GLU267. Notably, the score between Dp-3G and XO was −11.453 (Fig. 4D, E). Docking scores between targets on HUA-related metabolic pathways and corresponding chemicals are shown in Table S12-S13. Notably, the docking of Dp-3G with XO yielded the lowest score (−11.453), highlighting the notion that XO is a promising target for Dp-3G's therapeutic effect on HUA from a comprehensive view.

### Integration of molecular docking, MD simulation and SPR experiment confirms Dp-3G binding to XO

#### Validation of Dp-3G binding to XO through MD simulation

**Fig. 5:**
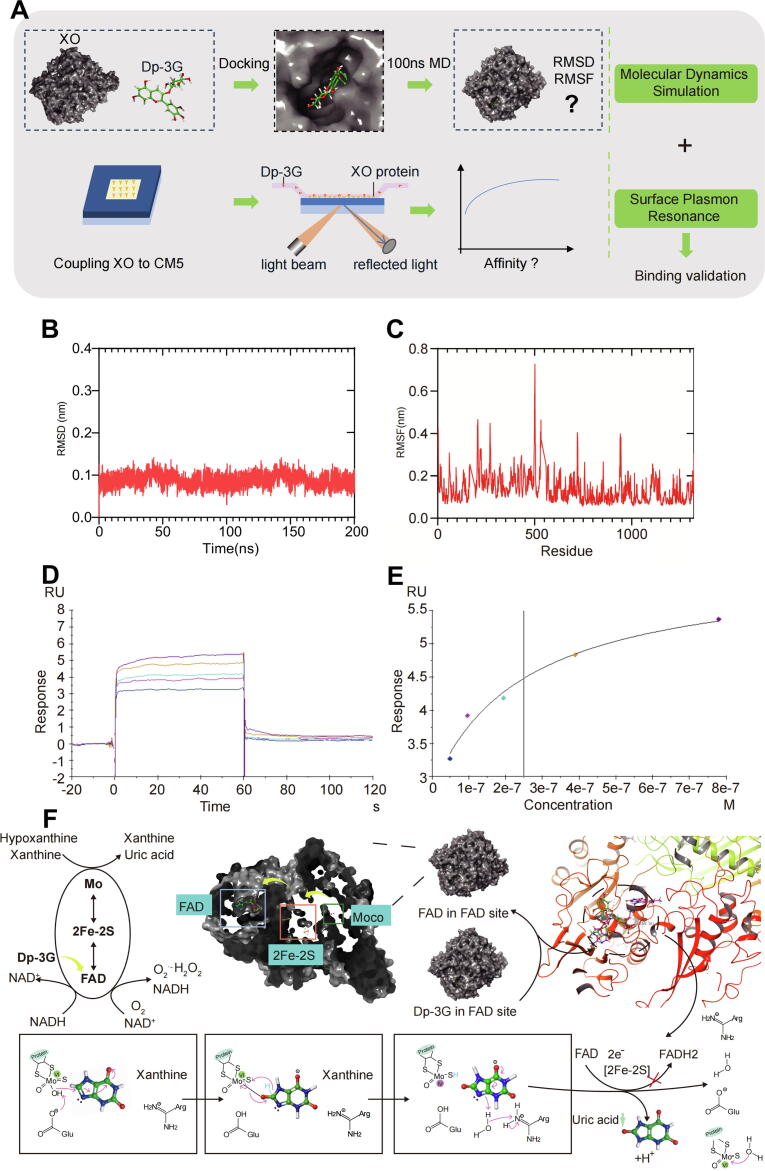
Verification of Dp-3G binding to XO and exploration of its impact on XO catalytic mechanisms. (A) Due to the direct correlation between XO and uric acid production, and the low binding score of Dp-3G with XO, integration of molecular dynamics simulations and surface plasmon resonance was employed to confirm the binding of Dp-3G to XO. (B) RMSD from molecular dynamics simulations of XO and Dp-3G within 200 nm. (C) RMSF from molecular dynamics simulations of XO with Dp-3G. RMSD and RMSF values indicate stable binding of XO to Dp-3G in molecular dynamics simulations. (D) Binding sensorgram showing the interaction between XO and Dp-3G (0.78, 0.40, 0.20, 0.10, 0.05 µM) in surface plasmon resonance. (E) Steady-state analysis plot of XO and the small molecule Dp-3G. Surface plasmon resonance experiments demonstrates moderately strong affinity between XO and Dp-3G. (F) Overview of XO catalytic mechanism in oxidizing hypoxanthine to xanthine, and xanthine to uric acid via Moco, [2Fe-2S], and FAD. Binding of Dp-3G to XO blocks uric acid production by occupying the FAD site. Abbreviations: RMSD: Root Mean Square Deviation; RMSF: Root Mean Square Fluctuation.

Due to the direct correlation between XO and uric acid, alongside the notably low docking score observed between Dp-3G and XO, we identified Dp-3G binding to XO as a primary target for further investigation. MD simulation offers clearer insights into the dynamics of interacting molecules over time, providing significant complementary information to docking studies and revealing conformational states relevant to more realistic environments [72]. Consequently, the complex structure of XO-Dp-3G obtained from docking was subjected to a 200 ns MD simulation, demonstrating the stability of the Dp-3G and XO combination in a simulated aqueous environment (Fig. 5A). Root mean square deviation (RMSD) in protein–ligand complexes from a 200 ns MD simulation (Fig. 5B) shows that the RMSD remained approximately 0.1 nm, indicating the XO enzyme-ligand complex achieved a stable conformation throughout the MD simulation. Additionally, the root mean square fluctuation (RMSF) of the XO enzyme throughout the entire 200 ns MD simulation shows that residues surrounding the protein's active site stabilized after the Dp-3G structure bound to the interior of the enzyme (Fig. 5C and Movie S1). This observation suggests that the binding of Dp-3G may impact the XO enzyme's active site, potentially influencing its catalytic function.

#### SPR experiment indicates moderately strong binding of Dp-3G to XO enzyme

To further validate and determine the binding affinity between Dp-3G and XO, a SPR measurement was conducted in this study (Fig. 5A). Fig. 5D illustrates the dynamic process of Dp-3G binding to XO at various concentrations, indicating a steady state of binding. The maximum binding response increased gradually with concentration until reaching saturation. Around 60 s, the small molecules were removed, and the response units decreased rapidly, indicating gradual dissociation of Dp-3G from the XO surface.

Fig. 5E presents the results of the steady-state analysis, illustrating the relationship between RU at equilibrium and Dp-3G concentration. By fitting this curve, we determined a dissociation constant (KD) of 0.25 μM (2.5 × 10^−7^ M) for the interaction between Dp-3G and XO. This KD value falls within the range of 10^−7^ M, indicating a moderately strong binding affinity between Dp-3G and XO, which ensures efficient interaction without leading to irreversible binding and potential toxicity. The results of the SPR experiment provide strong evidence of the direct interaction between Dp-3G and XO.

#### Participation of Dp-3G as an XOI by occupying the FAD binding site in XO enzyme

Based on our molecular docking and MD simulation results, as well as verification through the SPR experiment, we proceeded to investigate the precise role of Dp-3G in XO activity. Notably, we observed that Dp-3G occupies the FAD binding site in the XO protein, forming hydrogen bonds with both ASN261 and VAL259 residues.

In the normal catalytic cycle of the XO enzyme, electrons are transferred from substrates (hypoxanthine, xanthine) through the molybdopterin cofactor (Moco), [2Fe-2S], and finally to FAD, completing the oxidation reaction to produce uric acid [73,74]. However, upon binding to the FAD site, Dp-3G disrupts this electron transfer process. Specifically, Dp-3G interferes with the final step of electron transfer from [2Fe-2S] to FAD, thereby inhibiting XO's ability to catalyze uric acid production. This provides a molecular mechanistic explanation for Dp-3G as an XOI (Fig. 5F).

The comprehensive and integrated research approach we adopted, spanning from in silico analysis and simulation to experimental verification, yielded mutually supportive and validated results. This consistency across multiple methodologies enhances the credibility of our conclusions and robustly supports the hypothesis that Dp-3G acts as an XOI. These findings establish a strong foundation for further investigations into the biological activity and potential therapeutic applications of Dp-3G.

### Dp-3G not only reduces uric acid levels but also exerts other beneficial metabolic effects

#### Biochemical and cellular experiments confirm that Dp-3G decreases uric acid levels through inhibition of XO catalytic activity

**Fig. 6:**
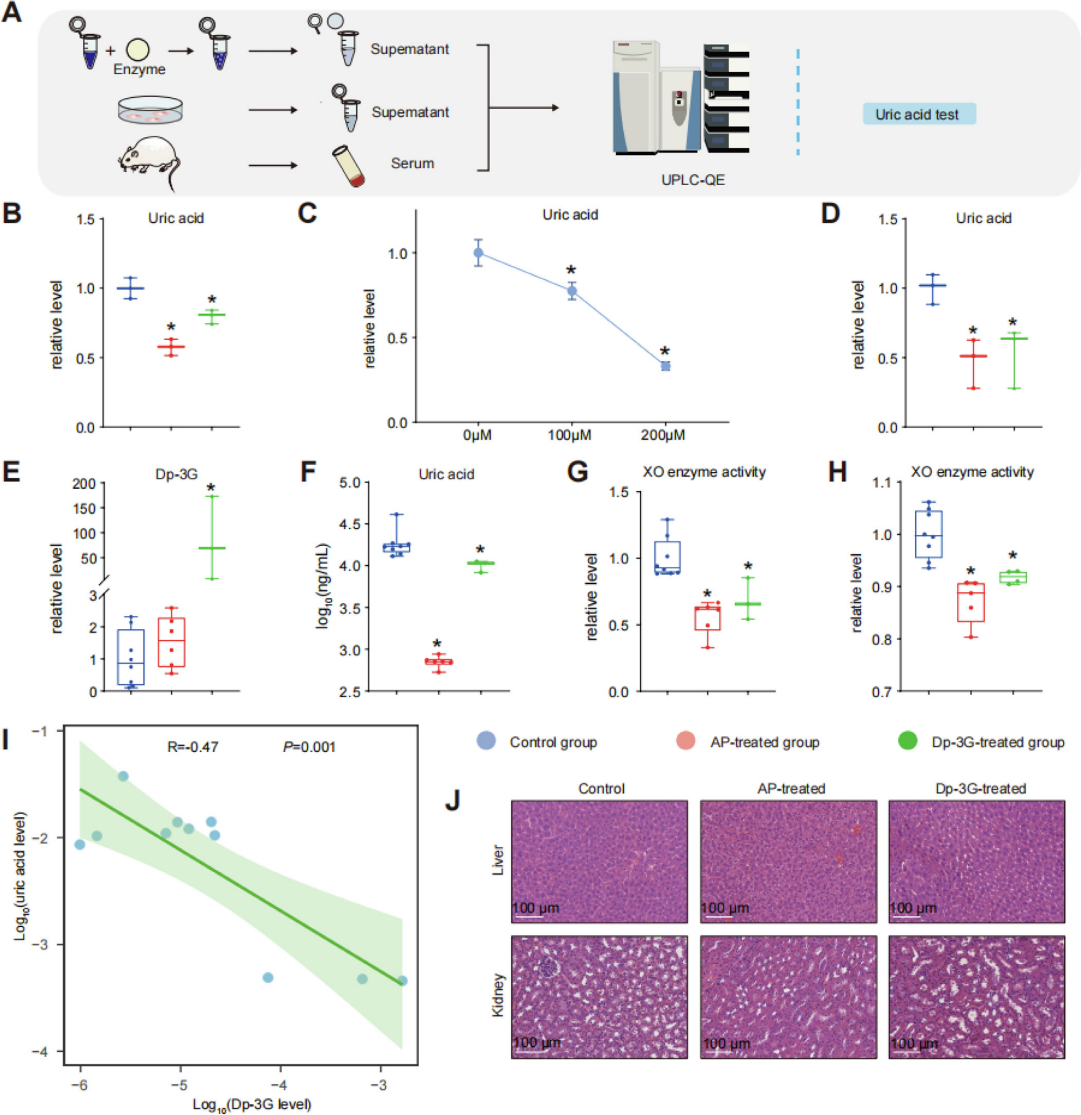
Effects of Dp-3G on uric acid production using biochemical and living organism experiments both *in vitro* and *in vivo*. (A) Design of XO reaction-UHPLC/MS coupling analysis and *in vitro* and *in vivo* metabolomic experiments. (B) Comparison of uric acid levels among the three groups in biochemical reaction experiments, indicating a decrease in uric acid with both AP and Dp-3G treatments (n = 3 biological replicates). (C) Effect of different concentrations of Dp-3G on uric acid levels in biochemical reaction experiments (n = 6 biological replicates, mean ± SEM). (D) Comparison of uric acid levels among the three groups in cell experiments, indicating a decrease in uric acid with both AP and Dp-3G treatments (n = 3 biological replicates). (E-F) Comparison of blood serum Dp-3G and uric acid levels among the three groups in the HUA mouse model (n = 8, 6, and 3 biological replicates for control, AP-treated, and Dp-3G-treated groups, respectively), showing decreased uric acid levels with both AP and Dp-3G treatments, and increased serum Dp-3G levels with Dp-3G treatment. (G) Effect of AP and Dp-3G treatment on XO enzyme activity *in vivo* during intraperitoneal injection experiments (n = 8, 6, and 3 biological replicates for control, AP-treated, and Dp-3G-treated groups, respectively). (H) Effect of AP and Dp-3G treatment on XO enzyme activity *in vivo* during oral gavage experiments (n = 8, 5, and 4 biological replicates for control, AP-treated, and Dp-3G-treated groups, respectively). Relative content is represented in box plots. The central horizontal line indicates the median value, while the upper and lower bounds denote the maximum and minimum values. The top and bottom edges of the box correspond to the 75th and 25th percentiles, respectively. * indicates significance compared to the control group, with *P* < 0.05. (I) Dose-response relationship between Dp-3G and uric acid in mouse serum, demonstrating a dose-dependent decrease in serum uric acid with increasing Dp-3G concentration. (J) H&E staining of liver and kidney tissues, indicating no pathological changes caused by AP or Dp-3G treatments. Bar = 100 μm. Abbreviations: Dp-3G: delphinidin-3-glucoside, AP: allopurinol.

To further investigate the inhibition of XO by Dp-3G in reducing uric acid levels, we conducted biochemical reaction experiments using the XO reaction-UHPLC coupling method (Fig. 6A). We observed a significant reduction in uric acid levels in the AP treated group, a potent purine-like XOI and classical drug for HUA. Similarly, a decrease in uric acid levels was also noted in the Dp-3G treated group (Fig. 6B-C).

Subsequently, we examined the inhibition of XO by Dp-3G in reducing uric acid levels in living organisms *in vitro* (Fig. 6A). Consistently, we observed a significant reduction in uric acid levels in the AP treated group, and a decrease was also evident in the Dp-3G treated group (Fig. 6D).

#### Dp-3G lowers uric acid levels in mice

**Fig. 7:**
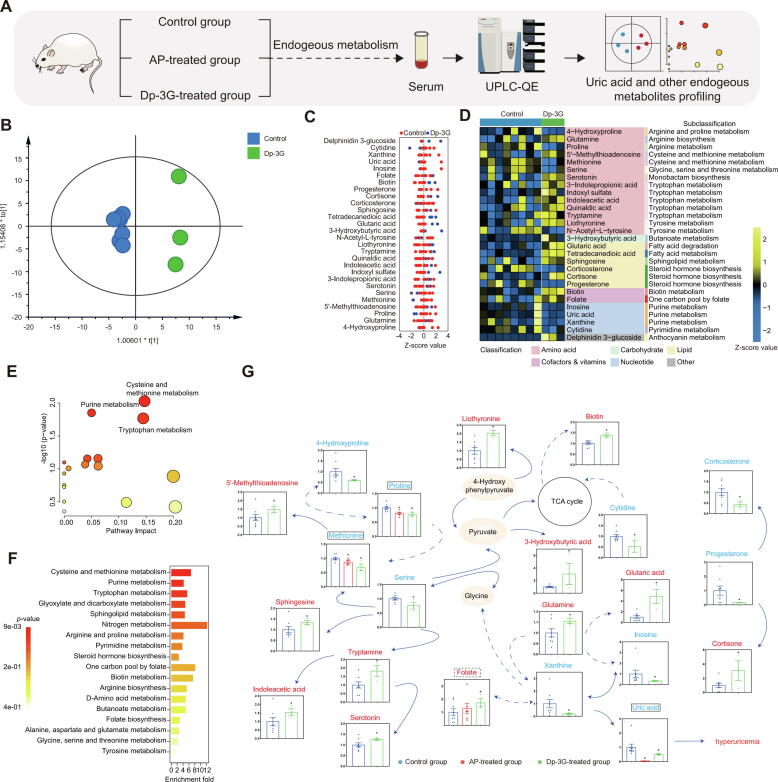
Results of metabolomic experiments in the HUA mouse model. (A) Design of mouse serum metabolomic experiments. (B) OPLS-DA of metabolomes between the control and Dp-3G-treated groups. R2Y(cum) and Q2(cum) were 0.990 and 0.452, respectively, indicating global metabolic changes caused by Dp-3G treatment. (C) Z-score plot depicting differential metabolites between the control and Dp-3G-treated groups. (D) Heatmap illustrating differential metabolites between the control and Dp-3G-treated groups. (E-F) Pathway analysis and enrichment analysis of differential metabolites between the control and Dp-3G-treated groups, respectively. (G) Integrated metabolic network analysis of differential metabolites between the control and Dp-3G-treated groups. Metabolites exhibiting decreased levels in the Dp-3G-treated group compared to the control group are depicted in blue, while those showing increased levels are indicated in red. Within the square boxes, bar graphs illustrate the relative level comparison of these metabolites across groups. * indicates significance compared to the control group, with P < 0.05. The data are shown as mean ± SEM (n = 8, 6, and 3 biological replicates for control, AP-treated, and Dp-3G-treated groups, respectively). Metabolite names enclosed in dashed-line boxes signify the reversal of HUA-associated metabolic alterations including folate reported in the literature [77] by Dp-3G, whereas those in solid-line boxes denote reversal including methionine and proline by both interventions.[75,76,78] Abbreviations: Dp-3G: delphinidin-3-glucoside, AP: allopurinol. (For interpretation of the references to color in this figure legend, the reader is referred to the web version of this article.)

Subsequently, we established a mouse model of HUA and treated it with AP and Dp-3G (Fig. 7A and Fig. S10A). Following administration of Dp-3G, a significant increase in Dp-3G levels was observed compared to both the HUA model and AP-treated groups, indicating successful Dp-3G treatment and the presence of the parent compound in the liver for XO binding (Fig. 6E).

HUA is characterized by elevated uric acid levels. As shown in Fig. 6F, serum uric acid levels were significantly lower in both the AP-treated and Dp-3G-treated groups compared to the HUA model group. The conclusion remained valid even after excluding the high value in the control group (Control *vs*. Dp-3G-treated, *P* = 0.02; Control *vs*. AP-treated, *P* < 0.01). Additionally, XO activity in liver extracts was assessed across all groups. XO activity was significantly reduced in both the Dp-3G-treated and AP-treated groups (Fig. 6G). In addition, Dp-3G and AP significantly suppressed the activity of XO enzyme in liver after oral administration (Fig. 6H), highlighting an inhibitory effect of the Dp-3G on XO activity *in vivo*. Furthermore, serum uric acid levels decreased dose-dependently with increasing concentrations of Dp-3G (R = -0.47, *P* = 0.001) (Fig. 6I). These findings collectively indicate that Dp-3G can reduce uric acid levels in biochemical, *in vitro*, and *in vivo* settings.

#### Effects of Dp-3G on various endogenous metabolisms related to HUA-induced diseases

As depicted in Fig. S10B-E, there were no statistically significant differences in body weight among the groups. Additionally, there were no significant changes observed in liver and kidney organ coefficients (%), nor in liver and kidney morphology as revealed by hematoxylin and eosin (H&E) staining (Fig. 6J).

To systematically elucidate the endogenous metabolic changes underlying the therapeutic effects of Dp-3G in the HUA mouse model, we analyzed metabolomic alterations between the HUA model and Dp-3G-treated groups. To assess systemic changes in the metabolome of HUA mice treated with Dp-3G, an OPLS-DA model was employed to explore the differences in metabolic profiles between the two groups. The resulting R2Y(cum) and Q2 (cum) values of 0.990 and 0.452, respectively, indicate a good fit and predictability of the OPLS-DA model (Fig. 7B). These findings suggest distinct metabolic phenotypes between the HUA model and Dp-3G-treated groups.

**Fig. 8:**
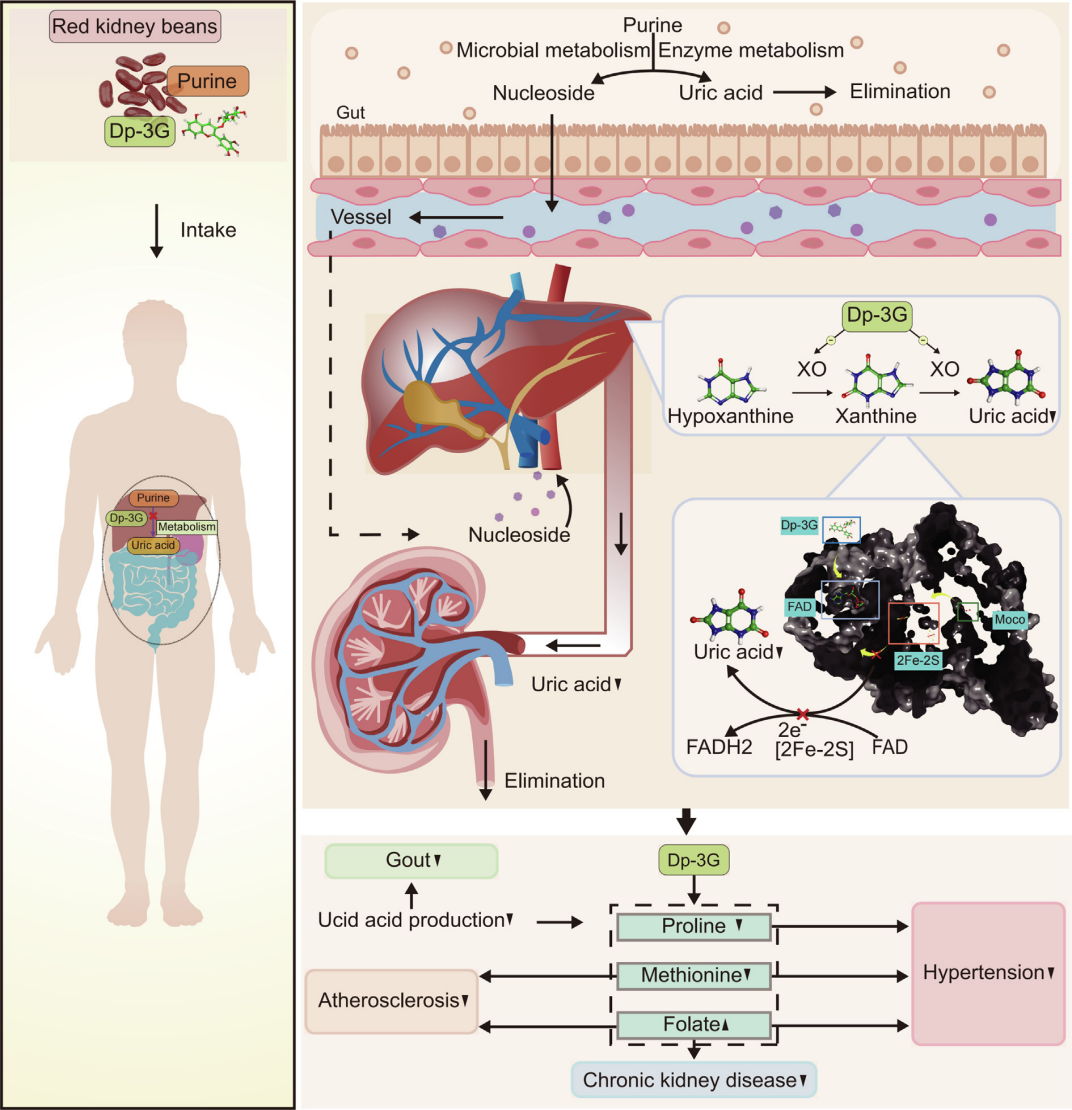
Mechanism underlying the decrease in uric acid through intake of red kidney beans. Dp-3G, abundant in red kidney beans, functions as a xanthine oxidase inhibitor. It binds to the XO enzyme, thereby blocking the generation of uric acid by occupying the FAD binding site within the XO enzyme and disrupting the electron transfer chain (Moco, [2Fe-2S], FAD) in the purine metabolic pathway. This mechanism reduces the risk of HUA and contributes to the reversal of other metabolic changes associated with HUA, such as alterations in methionine, proline, and folate. These changes are linked to complications of HUA, including atherosclerosis, hypertension, and chronic kidney disease. (For interpretation of the references to color in this figure legend, the reader is referred to the web version of this article.)

Compared with the HUA model group, 28 metabolites were found to be altered in the Dp-3G-treated group (Fig. 7C). We subsequently examined the biological connections of these differential metabolites and classified them into metabolic pathways (Fig. 7D). The decrease in xanthine alongside uric acid, and the unchanged levels of hypoxanthine, collectively demonstrate that Dp-3G reduces uric acid through XO [39] (Fig. 3D and Fig. S9). Pathway enrichment analysis revealed that the differential metabolites between the HUA model and Dp-3G-treated groups were primarily enriched in “Cysteine and methionine metabolism”, “Purine metabolism”, and “Tryptophan metabolism” (Fig. 7E, F and Table S14-S15). Furthermore, metabolite network integration analysis (Fig. 7G, Fig. S10F) comprehensively integrated all differential metabolites between the two groups into a metabolic network, elucidating the underlying connections of the overall serum metabolic alterations induced by Dp-3G treatment. Our findings indicate that Dp-3G can reverse metabolic alterations associated with HUA as reported in the literature, including changes in methionine, proline, and folate [[75], [76], [77], [78]]. Through a literature review to understand the implications of these metabolites in HUA-related diseases, we found that they are notably involved in the pathogenesis of such conditions. In comparison to the classical XOI drug AP, Dp-3G showed the ability to reverse alterations in metabolites affected by AP, such as methionine and proline. Dp-3G also affected metabolites that AP cannot significantly alter, such as folate (Table S16). These findings suggest additional metabolic benefits of Dp-3G due to its influence on metabolites involved in the pathogenesis of HUA-related diseases (Fig. 8).

## Discussion

XO is an enzyme primarily involved in the catabolism of purine nucleic acids [39]. Currently, the identification and development of agents that bind to XO (XOI) are the primary sources for ULT. Two approved drugs in the US include purine-like AP, which is the cornerstone of treatment for patients with HUA, and non-purine-like febuxostat, which serves as an alternative to AP. The 2020 American College of Rheumatology (ACR) and 2016 European League Against Rheumatism (EULAR) guidelines recommend low-dose AP as the first-line treatment for gout associated with HUA [79]. However, these drugs may cause serious side effects [6], prompting a search for safe and effective natural XOIs, which has become a focus of research.

The relationship between legumes and HUA has drawn attention, and discrepancies in human evidence may arise from the various types of legumes and their diverse natural compounds, as well as differences in population susceptibility [80]. Some studies have indicated that consuming red kidney beans can reduce the risk of cardiovascular disease [81]. Interestingly, HUA itself can induce cardiovascular disease [2]. Our study discovered that red kidney beans may lower uric acid levels, suggesting that their beneficial vascular effects might be partially attributed to their urate-lowering components.

Anthocyanins, widely recognized as flavonoid compounds, are water-soluble natural polyphenolic pigments found abundantly in fruits and vegetables [67]. Interestingly, specific anthocyanins such as delphinidin-3-O-sambubioside is now considered to be XOI [82]. However, the effect of Dp-3G on HUA has not been previously reported. This study reveals for the first time the potential of Dp-3G, found in red kidney beans, as a novel natural XOI. Dp-3G has also been shown to confer health benefits [83], with studies demonstrating its presence as a parent compound following food processing and in the ADME processes within the human body [84,85]. Existing studies offer valuable insights into the absorption and distribution of Dp-3G following oral administration. Research indicates that anthocyanins, including Dp-3G, are absorbed as parent compound and quickly transported to the liver without structural alteration [86]. Long-term ingestion studies, mimicking human daily diets, show that the parent compound of Dp-3G remains the predominant form in the body, with no significant presence of aglycans or conjugates in plasma [[44], [45], [46]]. Therefore, Dp-3G reaches the liver in its parent form after oral intake, where it binds to the XO enzyme to reduce uric acid synthesis. Each catalytically independent subunit of XO includes [2Fe-2S] in the N-terminal domain, FAD cofactor in the intermediate domain, and Moco in the C-terminal domain [87]. Leveraging human evidence and an integrated C-T-M network, along with biosensor, biochemical, cellular, and murine studies, we comprehensively elucidated the mechanism by which Dp-3G exerts its XOI effect through moderately strong binding to XO and occupation of the FAD site. Molecular docking results demonstrated a binding score of −11.453 for Dp-3G to XO, indicating a moderately strong binding affinity. This finding was further validated through SPR experiment, which yielded a measured KD value of 0.25 μM. Importantly, we discovered that Dp-3G occupies the FAD site, disrupting the electron transport chain between Moco, [2Fe-2S], and FAD, thus effectively inhibiting XO activity and uric acid production.

Multiple studies have demonstrated that individuals with higher BMI, such as overweight individuals with type 2 diabetes [88], show increased XO activity. This aligns with findings from a Malaysian study on human plasma XO activity, which reported a positive correlation between XO activity and body weight [89]. These results underscore the importance of weight management in controlling HUA and lend further support to our discovery that consuming Dp-3G-rich red kidney beans may have a more pronounced effect in reducing HUA risk among obese individuals. This information helps explain the varying susceptibility of red kidney beans' effects on HUA within the studied population. Overall, our study highlights red kidney beans for intervening in HUA through the XOI mechanism.

In our study, we observed that the binding strength between XO and Dp-3G was moderate strong, and such binding would not lead to irreversible binding and potential toxicity. Furthermore, using a HUA mouse model, we investigated the effects of Dp-3G beyond its uric acid-lowering properties. We did not observe any impact on liver and kidney coefficients or their histology, indicating no apparent toxicity. HUA, a primary risk factor for serious diseases such as chronic kidney disease, atherosclerosis, and hypertension, disrupts various metabolic processes including the oxidative hydroxylation of hypoxanthine to xanthine and uric acid, leading to reactive oxygen species generation via XO [2,73]. Our study revealed that Dp-3G significantly influences “Cysteine and methionine metabolism”, “Purine metabolism”, and “Tryptophan metabolism”. Furthermore, Dp-3G reversed metabolic abnormalities associated with HUA, including changes in methionine, proline, and folate [[75], [76], [77], [78]], showing effects similar to AP. Other researches have also shown that AP reduces serum methionine levels in HUA patients [90], and proline levels in HUA mouse models [91], indicating the XOI related metabolic changes. The disruption of methionine metabolism has been linked to the development of atherosclerosis in humans, animal models, and vascular smooth muscle cells. This disruption of methionine metabolism causes atherosclerosis through mechanisms such as increasing plasma lipid levels, disturbing lipid peroxidation and antioxidant processes, and causing dysfunction in endothelial cells and arterial smooth muscle cells [[92], [93], [94], [95]]. Moreover, excess methionine levels have been implicated in elevated blood pressure in humans, rats, and vascular smooth muscle cells *in vitro*. This effect is mediated by increased endothelin type B receptor-mediated contraction, activation of angiotensin-converting enzyme, and inhibition of nitric oxide synthesis, all contributing to elevated blood pressure [[96], [97], [98], [99]]. Proline has been implicated in increasing the risk of hypertension in humans, rats, and cultured rat vascular smooth muscle cells. This effect is mediated by influencing the release of vasopressin from the hypothalamus into the bloodstream, promoting collagen synthesis, and stimulating cell growth, thereby contributing to arterial remodeling [[100], [101], [102], [103]]. Folate has been shown to decrease the risk of chronic kidney disease in humans, mice, and human kidney proximal tubular cells by mitigating oxidative stress and reducing inflammation [[103], [104], [105], [106], [107]]. Additionally, folate supplementation has been linked to delayed development of atherosclerotic lesions in humans, mice, and human endothelial cell lines by influencing levels of monocyte chemoattractant protein 1 and modulating vascular endothelial growth factor DNA methylation [[108], [109], [110], [111]]. Folate can also help to keep normal blood pressure in humans, rats, and human pulmonary artery endothelial cells by enhancing nitric oxide bioavailability and improving endothelial function [[112], [113], [114], [115], [116], [117]]. The effects and underlying mechanisms of Dp-3G and AP-altered metabolites including methionine, proline, and folate on diseases including atherosclerosis, hypertension, and chronic kidney disease in humans, animals, and cells are summarized (Table S16). These results suggested additional metabolic benefits of Dp-3G in diseases linked to HUA with XOI mechanism involved.

DP-3G demonstrates superior advantages in terms of XOI effect and safety when compared to other natural products. In this study, the XO docking score for Dp-3G is exceptionally low (−11.453), lower than that of Cy-3G and other flavonoids, including hesperetin, urolithin, dihydromyricetin, and 3-O-methylquercetin [118,119]. MD simulations further demonstrated that Dp-3G forms an exceptionally stable complex with XO, maintaining a consistently low RMSD of 0.1 nm throughout the simulation, which is lower than that of other polyphenols and flavonoids, such as quercetin, kaempferol, isorhamnetin, and chlorogenic acid [120]. Moreover, our MD results indicate that compared to other polyphenolic compounds such as quercetin, kaempferol, isorhamnetin, and chlorogenic acid, as well as hesperetin, Dp-3G reaches a stable state more rapidly, further supporting its lower RMSD and faster stabilization. SPR results reinforce these findings, with Dp-3G exhibiting a KD of 0.25 μM, significantly outperforming other flavonoids, whose KD values range from 4.8 μM to 47.6 μM [121]. The combined docking, MD simulation, and SPR data strongly support Dp-3G as a highly potent XO inhibitor, exhibiting exceptional binding affinity, stability, and rapid conformational adaptation, positioning it as a more effective candidate for XO inhibition compared to other flavonoids and polyphenols. Regarding safety, no toxicity of DP-3G was observed in our study, as evidenced by normal organ coefficients, and its safety have been documented in the literature [46]. However, other urate-lowering flavonoids, such as daidzein, genistein and secoisolariciresinol, have been reported to be associated with potential male fertility toxicity [122]. Baicalein, a flavonoid, induces prolonged estrus and reduced fertility upon prenatal exposure in female mice [123]. Green tea polyphenols have been reported to induce potential nephrotoxicity and hepatotoxicity [124,125]. Our results also highlight the additional metabolic benefits of Dp-3G in managing HUA-related diseases with XOI mechanism involved. However, such metabolic reversals have not been reported for other natural products [126,127].

The study is limited by the case-control nature of the human population study for causality examination. However, we have implemented a series of statistical steps to enhance the scientific rigor of human data analysis and verify causal associations through the integration of molecular docking, MD simulations, SPR experiments, as well as biochemical, *in vitro*, and *in vivo* studies. Future randomized controlled clinical trials are needed to further validate and facilitate the translation of these findings into clinical applications in humans.

In conclusion, based on the C-T-M network, Dp-3G was identified as the key XO inhibitor in red kidney beans. Dp-3G consistently reduces uric acid production through the integration of molecular docking, MD simulations, SPR experiments, as well as biochemical, *in vitro*, and *in vivo* models, while also reversing HUA-related metabolic abnormalities in mice, including those involving methionine, proline, and folate. Our study identifies Dp-3G, a novel natural compound enriched in red kidney beans, which can disrupt the normal electron transfer process by binding moderately strongly to the FAD site on XO. This interference inhibits uric acid synthesis and reduces the risk of HUA, while also offering potential beneficial metabolic effects. The novel C-T-M network framework introduced in this study shows promise in studies of environmental and metabolic diseases. Furthermore, our findings significantly advance our understanding of edible red kidney beans and their natural component Dp-3G as a novel and potent inhibitor of the XO enzyme in the management of HUA.

## Compliance with Ethics Requirements

*All Institutional and National Guidelines for the care and use of animals (fisheries) were followed.*

## Compliance with Ethics Requirements

*All procedures followed were in accordance with the ethical standards of the responsible committee on human experimentation (institutional and national) and with the Helsinki Declaration of 1975, as revised in 2008 (5). Informed consent was obtained from all patients for being included in the study.*

## CRediT authorship contribution statement

**Yanling Chen:** Writing – original draft, Data curation, Investigation, Visualization, Formal analysis. **Yingtong Jiang:** Writing – original draft, Data curation, Investigation, Visualization, Formal analysis. **Lei Huang:** Data curation, Investigation, Visualization, Formal analysis. **Ziyi Li:** Validation, Software. **Mengyuan Zhu:** Validation, Visualization. **Lu Luo:** Writing – original draft, Investigation. **Kun Zhou:** Writing – original draft, Methodology. **Minjian Chen:** Writing – review & editing, Conceptualization, Funding acquisition, Project administration, Resources, Supervision.

## Declaration of competing interest

The authors declare that they have no known competing financial interests or personal relationships that could have appeared to influence the work reported in this paper.
