# Supplementary material for: Urate-lowering effect of delphinidin-3-glucoside in red kidney beans via binding to the FAD site of the XO enzyme
Source: J Adv Res. 2025 Apr 18;80:555–75. doi: 10.1016/j.jare.2025.04.022 (PMC12869243; doi:10.1016/j.jare.2025.04.022)
Supplement: Supplementary Data 1 [file mmc1.docx]

Supplementary Materials for

**Urate-lowering effect of delphinidin-3-glucoside in red kidney beans via binding to the FAD site of the XO Enzyme**

**Table of contents**

| **Section/topic** | **Reported on page #** |
| --- | --- |
| Fig. S1 | 2 |
| Fig. S2 | 3-4 |
| Fig. S3 | 5-6 |
| Fig. S4 | 7-8 |
| Fig. S5 | 9 |
| Fig. S6 | 10 |
| Fig. S7 | 11 |
| Fig. S8 | 12 |
| Fig. S9 | 13 |
| Fig. S10 | 14-15 |
| Table S1 | 16-18 |
| Table S2 | 19-21 |
| Table S3 | 22 |
| Table S4 | 23 |
| Table S5 | 24 |
| Table S6 | 25 |
| Table S7 | 26 |
| Table S8 | 27 |
| Table S9 | 28 |
| Table S10 | 29-30 |
| Table S11 | 31 |
| Table S12 | 32 |
| Table S13 | 33-34 |
| Table S14 | 35 |
| Table S15 | 36 |
| Table S16 | 37-44 |
| Movie S1 | 45 |
| Data S1 | 45 |
| References | 46-49 |

**Supplemental Figures and Figure Legends**


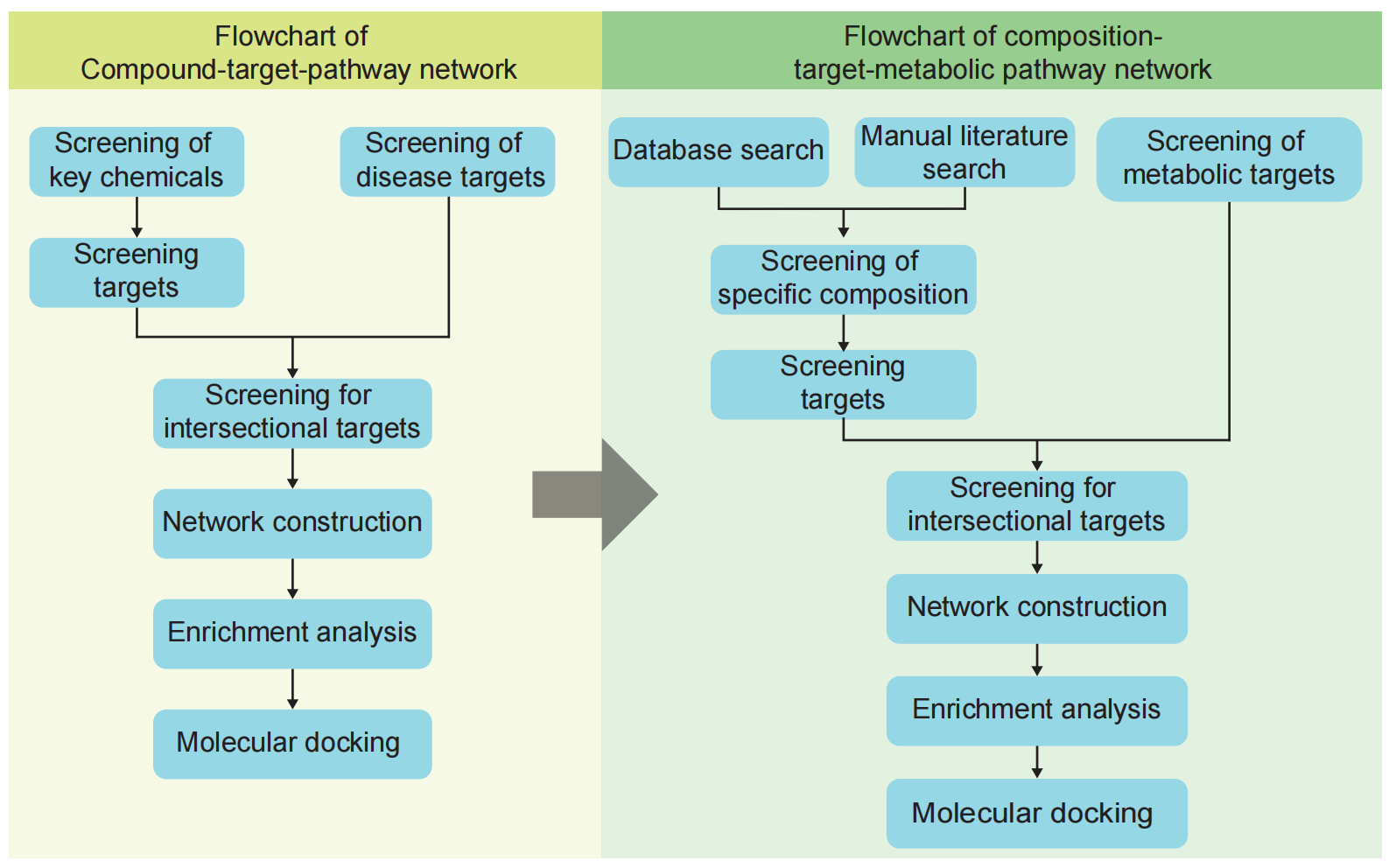


Fig. S1. A new C-T-M framework was established based on the principle of the C-T-P network framework to better suit the metabolic characteristics of HUA.


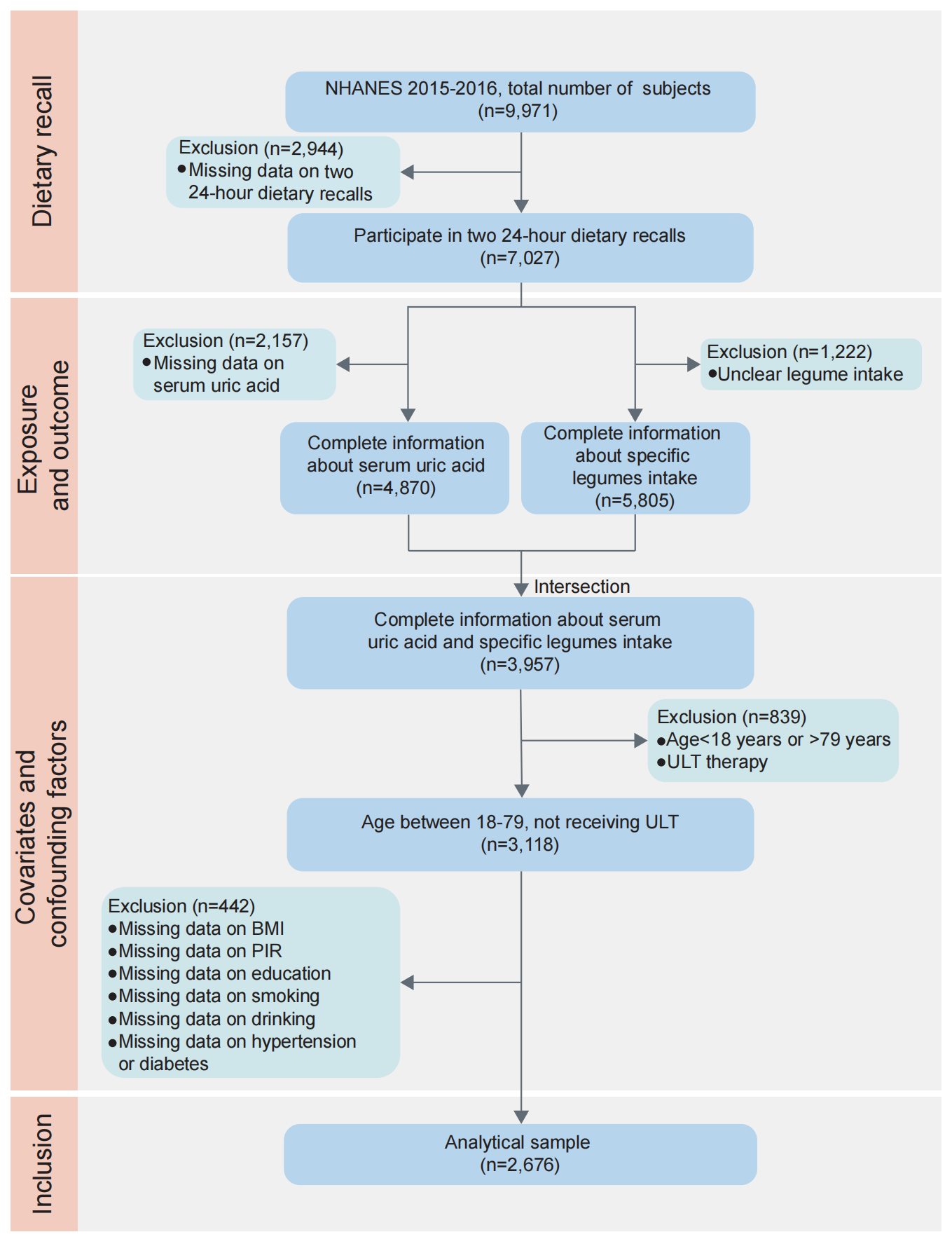


Fig. S2. Flow diagram illustrating participant inclusion and exclusion process. From the 9,971 participants in NHANES 2015-2016, eligible candidates were screened based on predefined inclusion and exclusion criteria, resulting in the final inclusion of 2,676 individuals in this study. Abbreviations: ULT: urate-lowering therapy; BMI: body mass index; PIR: poverty income ratio.


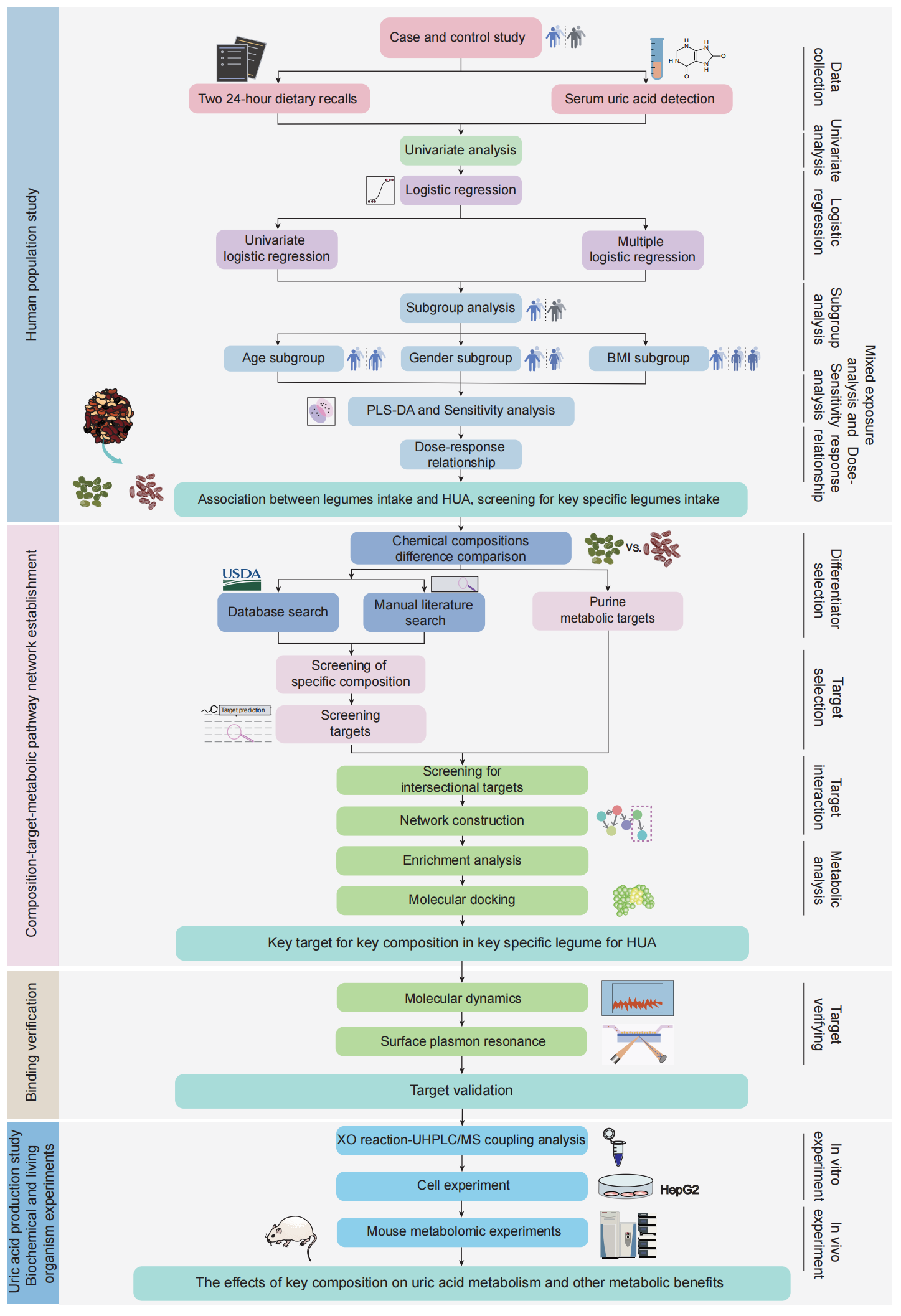


**Fig. S3.** Study design diagram. The study comprised four major steps, which included: human population study, establishment of specific chemical composition-target-pathway networks, verification of binding and uric acid production study using biochemical and living organism experiments.


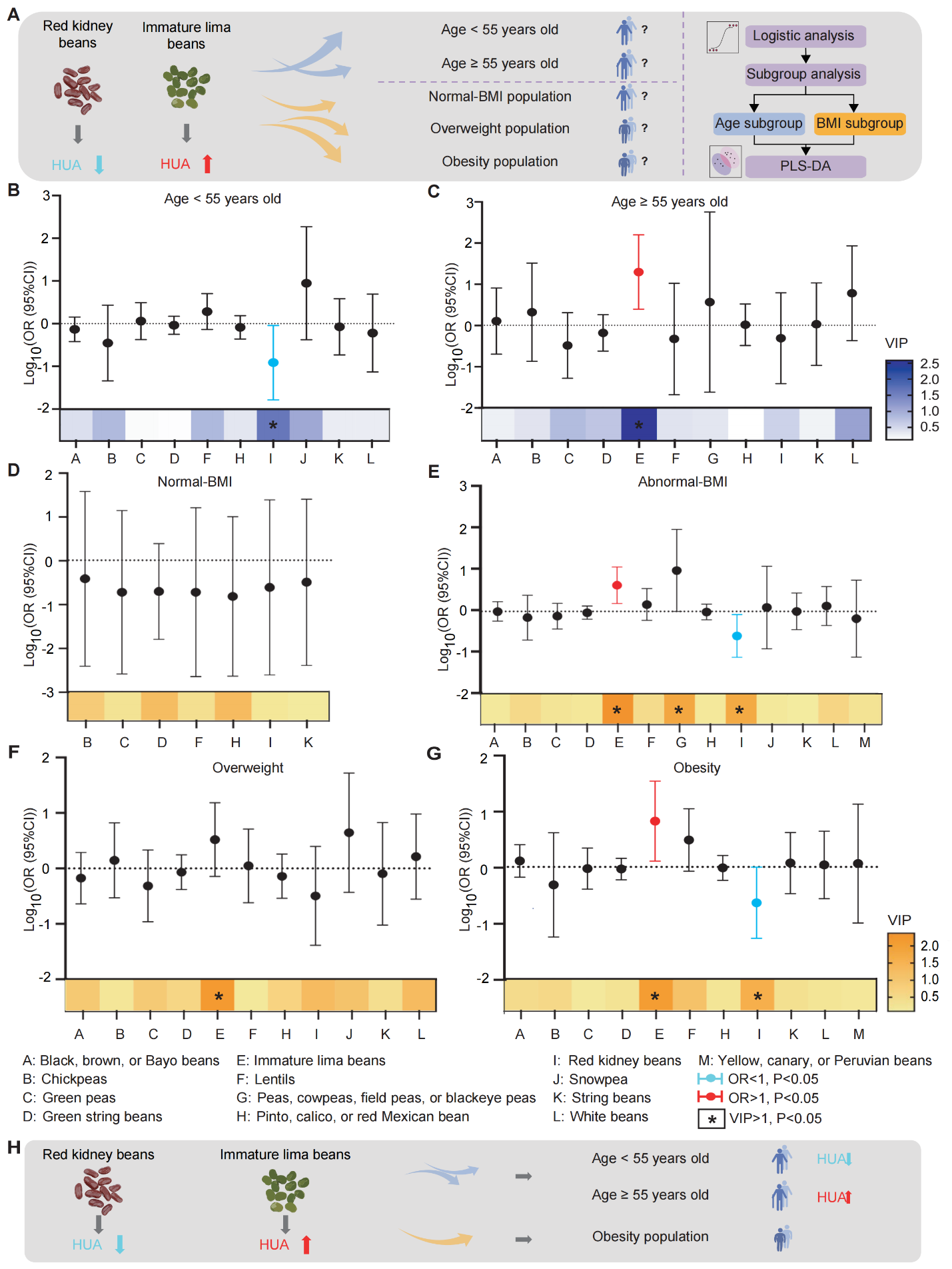


Fig. S4. Results of logistic regression and PLS-DA analyzing the relationship between immature lima beans and red kidney beans intake and HUA across age and BMI subgroups. (A) Study design focused on assessing the impact of legume intake on HUA across different age and BMI categories. (B-C) OR and 95% CI for the association of legume intake with the risk of HUA, alongside VIP scores from PLS-DA within age subgroups. (D-G) OR and 95% CI for the association of legume intake with the risk of HUA, alongside VIP scores from PLS-DA within BMI subgroups. (H) These findings highlight significant associations: immature lima beans in individuals aged 55 years or older, red kidney beans in individuals under 55 years old, and both immature lima beans and red kidney beans in obese individuals, all showing robust associations with HUA. Abbreviations: OR: Odds ratios; CI: confidence intervals; VIP: Variable Importance in Projection; PLS-DA: Partial Least Squares Discriminant Analysis.


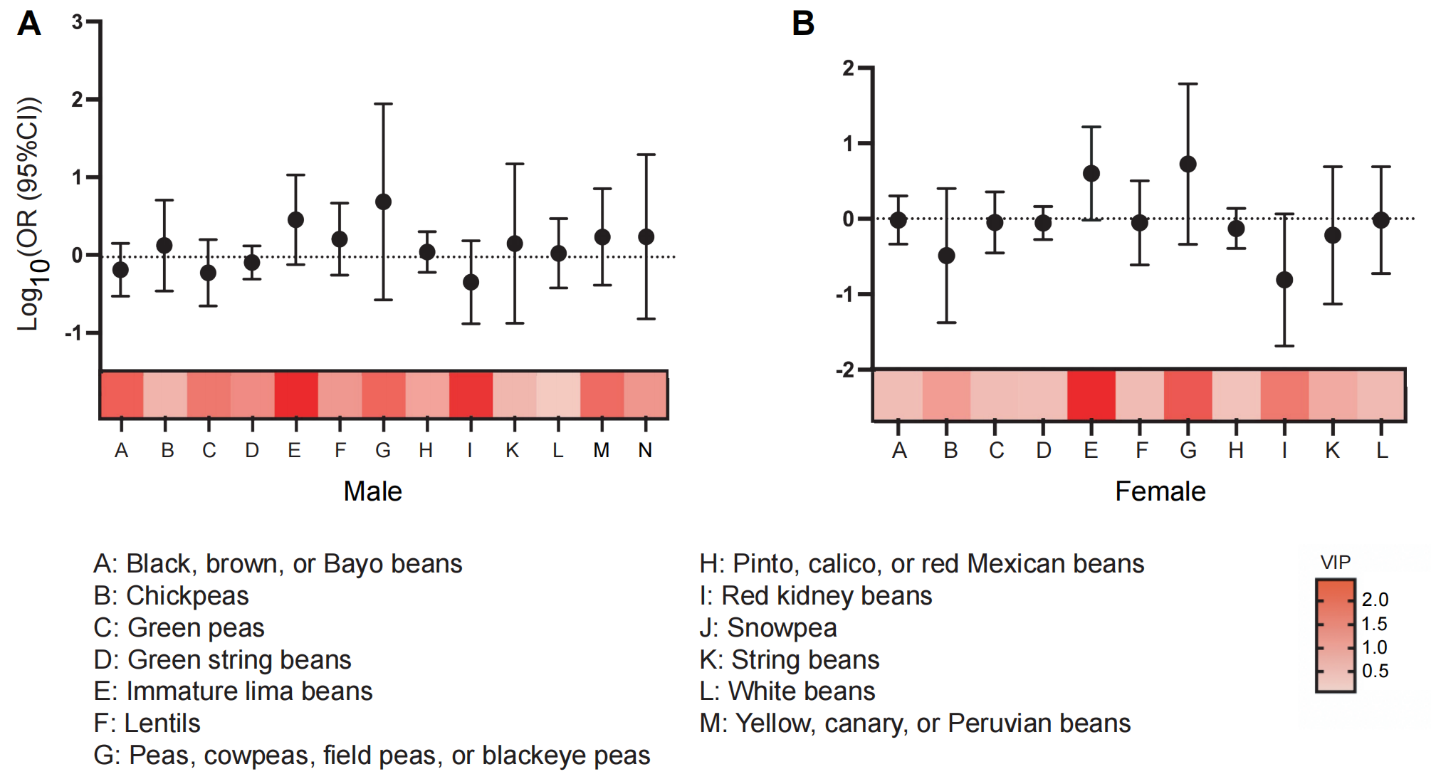


Fig. S5. Subgroup analysis by gender on the association between various legume intake rates and HUA.


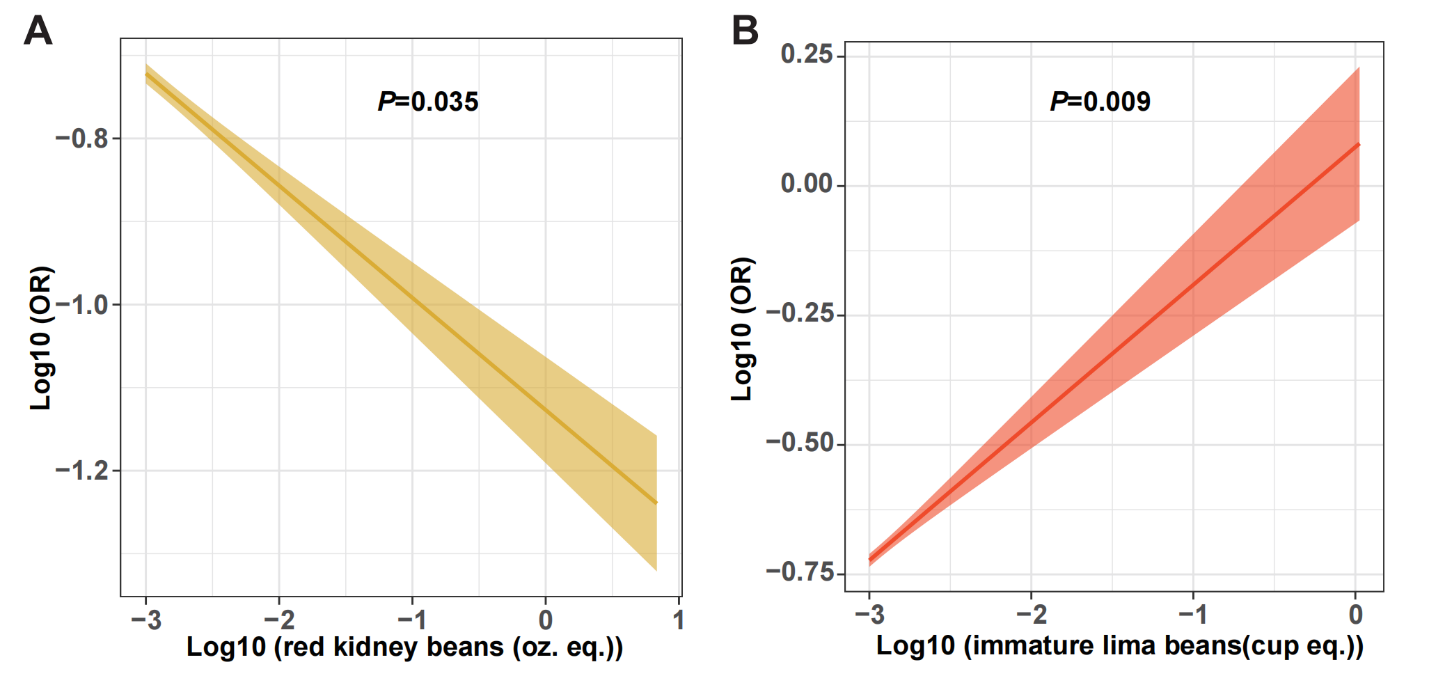


**Fig. S6.** The dose-response relationship between intake levels of red kidney beans and immature lima beans and the risk of HUA. Adjusted for gender, age, race, education, PIR, BMI, smoking, alcohol use, diabetes, and hypertension. A. Relationship between levels of red kidney beans intake and the risk of HUA. B. Relationship between levels of immature lima beans intake and the risk of HUA.


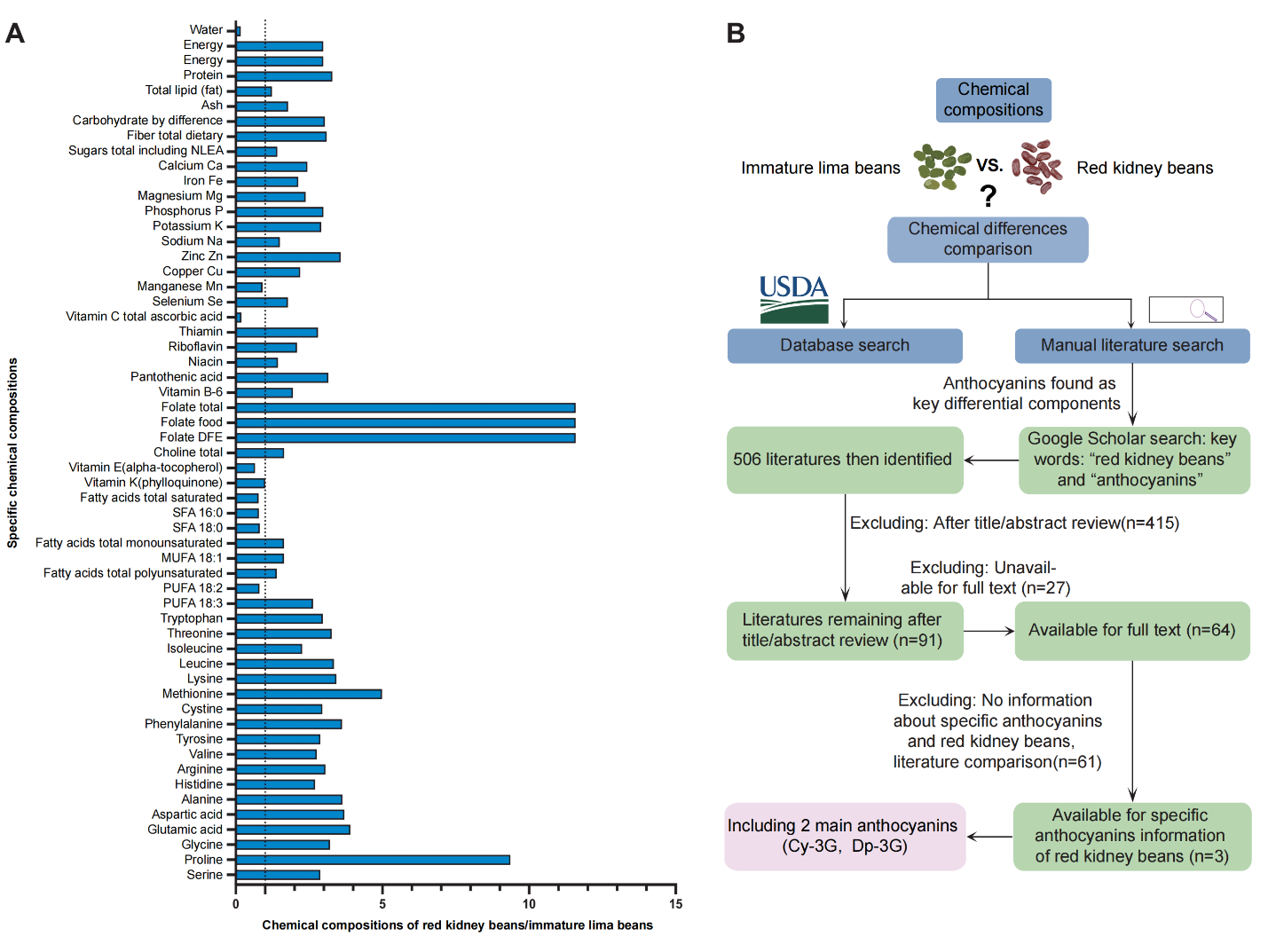


**Fig. S7.** Screening of specific compositions of key legumes. A. Ratio of components between red kidney beans (Beans, kidney, Red mature seeds, raw) and immature lima beans (Lima Beans, immature seeds, raw). Folate represents the most significant chemical composition that distinguishes between these two legumes. B. Manual literature screening process to identify specific anthocyanins in red kidney beans, revealing Cy-3G and Dp-3G as prominent anthocyanins. Abbreviations: Cy-3G: cyanidin-3-glucoside; Dp-3G: delphinidin-3-glucoside.


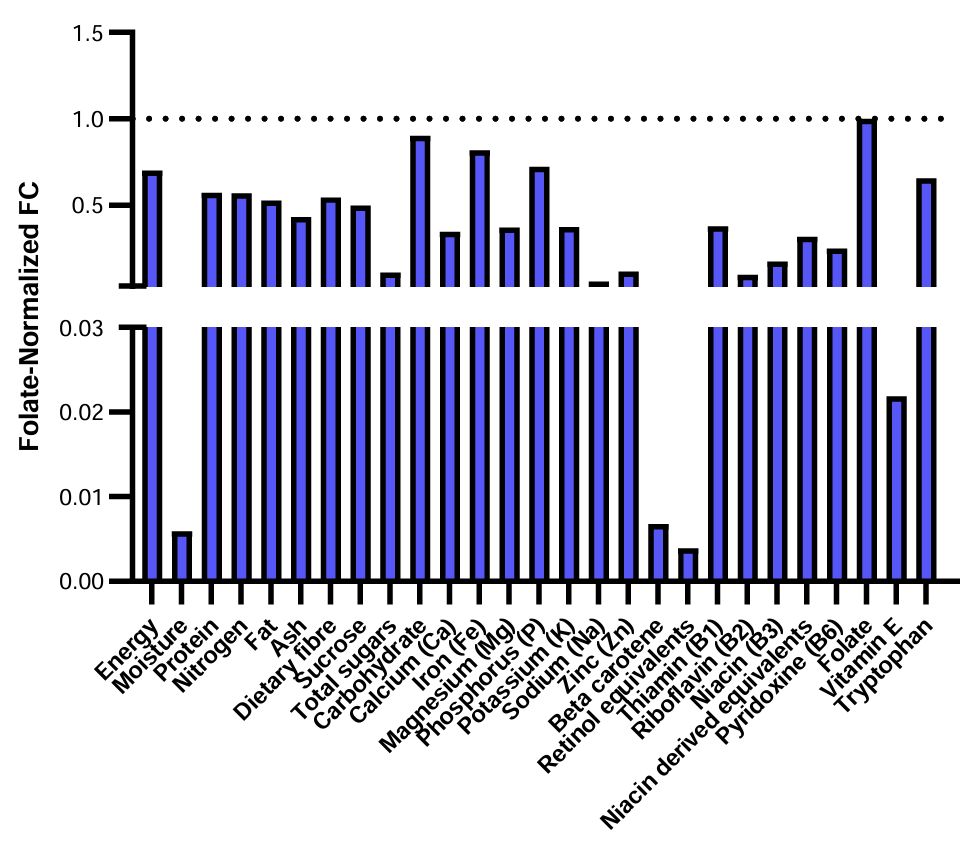


**Fig. S8.** Comparison of the composition of red kidney beans and lima beans using the Australian Food Composition Database.


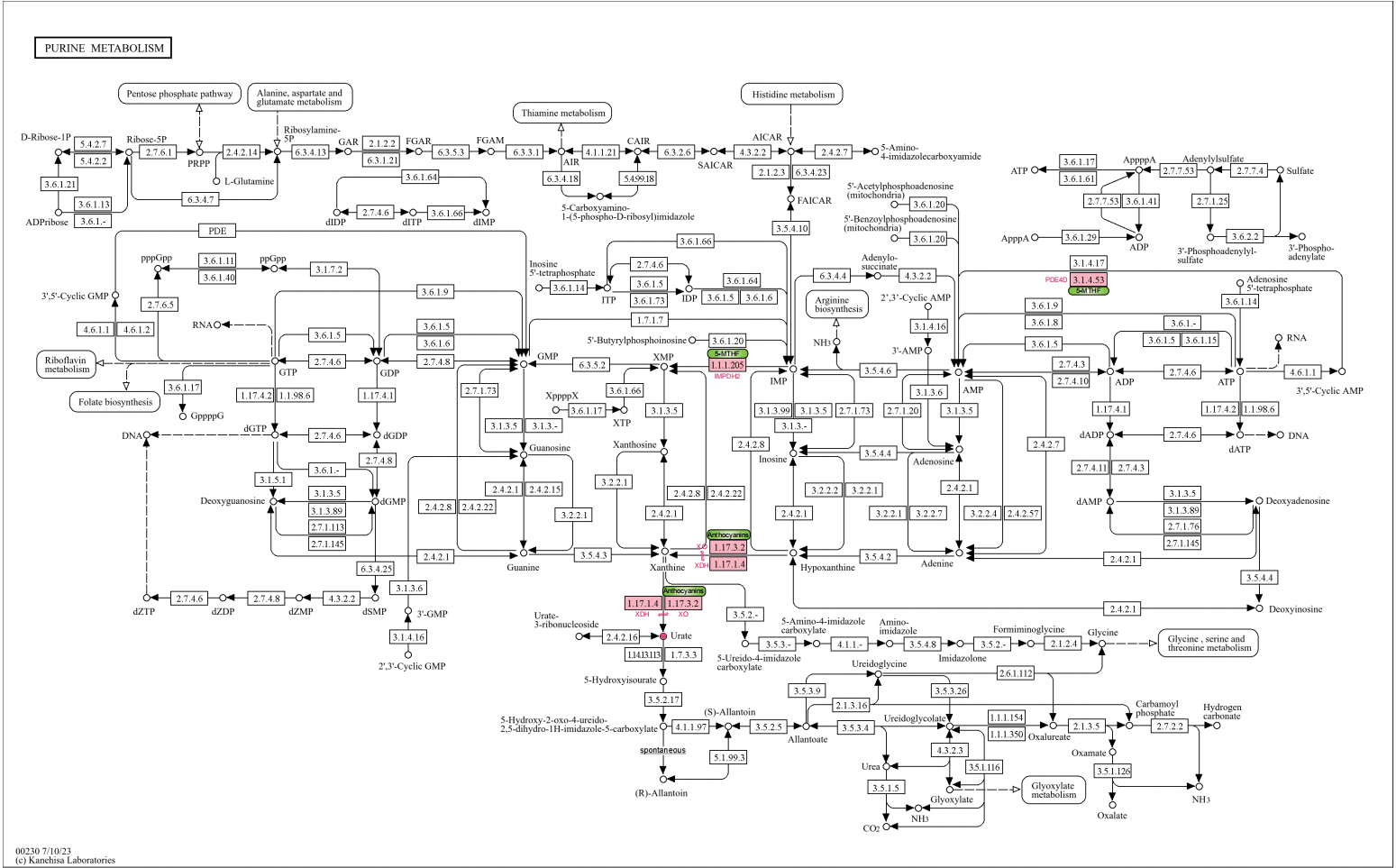


**Fig. S9.** Binding of 5-MTHF to IMPDH2 and PDE4D, and Dp-3G and Cy-3G to XO in the purine metabolic pathways as per the KEGG pathway database. Abbreviations: 5-MTHF: 5-Methyltetrahydrofolate; IMPDH2: Inosine-5'-monophosphate dehydrogenase 2; PDE4D: Phosphodiesterase 4D; XO: Xanthine oxidase.


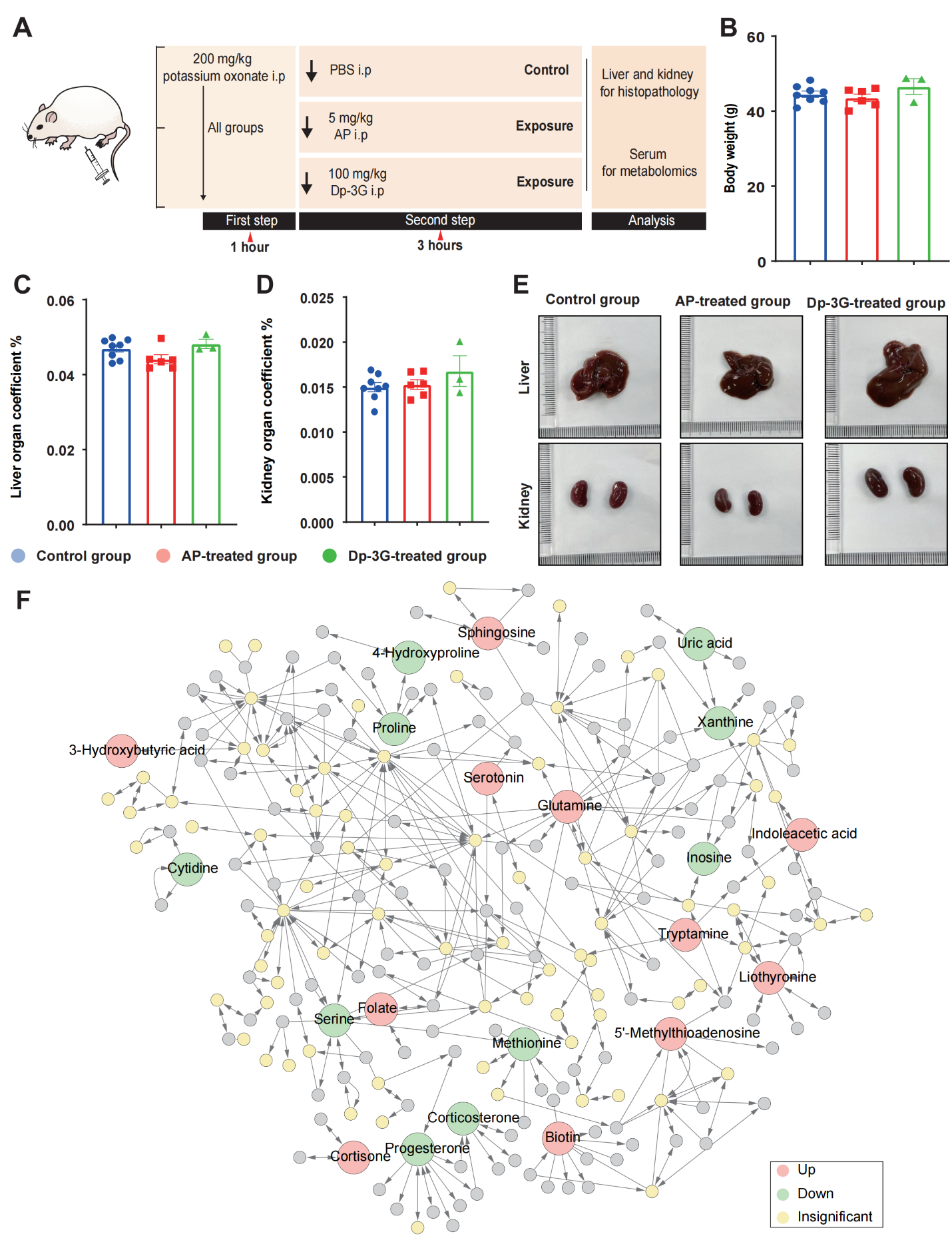


**Fig. S10.** Results of experiments in HUA mouse model. A. Schematic diagrams of animal experimental design. B. Body weight in each group shows no significant changes. C-D. Coefficient of liver and kidney in each group shows no significant changes. Data presented as mean ± SEM (n = 8, 6, and 3 biological replicates for control, AP-treated and Dp-3G-treated group, respectively). E. Representative images of liver and kidney of mice show no significant changes. F. Network of the metabolic changes analyzed by MetScape. Abbreviations: AP: allopurinol; Dp-3G: delphinidin-3-glucoside.

**Supplemental Tables**

Table S1. Basic characteristics of the study subjects and differences in these characteristics between the HUA and non-HUA groups.

|  | Level | Total Subjects N (%) or Mean (SD) | Non-HUA N (%) or Mean (SD) | HUA N (%) or Mean (SD) | *P* |
| --- | --- | --- | --- | --- | --- |
| N |  | 2,676 | 2,210 | 466 |  |
| Age  (Years) |  | 46.60 (17.13) | 45.73 (16.86) | 50.74 (17.79) | <0.001^*^ |
| Gender |  |  |  |  | 0.001^*^ |
|  | Male | 1,268 (47.4) | 1,013 (45.8) | 255 (54.7) |  |
|  | Female | 1,408 (52.6) | 1,197 (54.2) | 211 (45.3) |  |
| Race |  |  |  |  | 0.037^*^ |
|  | Non-Hispanic White | 927 (34.6) | 746 (33.8) | 181 (38.8) |  |
|  | Others | 1,749 (65.4) | 1,464 (66.2) | 285 (61.2) |  |
| Education |  |  |  |  | 0.303 |
|  | High school and below | 551 (20.6) | 464 (21.0) | 87 (18.7) |  |
|  | High school graduate/GED or equivalent | 664 (24.8) | 533 (24.1) | 131 (28.1) |  |
|  | Some college or AA degree | 793 (29.6) | 657 (29.7) | 136 (29.2) |  |
|  | College graduate or above | 668 (25.0) | 556 (25.2) | 112 (24.0) |  |
| PIR |  | 2.43 (1.59) | 2.44 (1.59) | 2.36 (1.57) | 0.273 |
| BMI (kg/m^2^) |  |  |  |  | <0.001^*^ |
|  | Abnormal | 1,995 (74.6) | 1,576 (71.3) | 419 (89.9) |  |
|  | Normal | 681 (25.4) | 634 (28.7) | 47 (10.1) |  |
| Smoking |  |  |  |  | 0.353 |
|  | Never smoked | 1,556 (58.1) | 1,294 (58.6) | 262 (56.2) |  |
|  | Smoked | 1,120 (41.9) | 916 (41.4) | 204 (43.8) |  |
| Alcohol use | |  |  |  | 0.912 |
|  | No | 831 (31.1) | 685 (31.0) | 146 (31.3) |  |
|  | Yes | 1,845 (68.9) | 1,525 (69.0) | 320 (68.7) |  |
| Hypertension | |  |  |  | <0.001^*^ |
|  | No | 1,684 (62.9) | 1,430 (64.7) | 254 (54.5) |  |
|  | Yes | 992 (37.1) | 780 (35.3) | 212 (45.5) |  |
| Diabetes |  |  |  |  | <0.001^*^ |
|  | No | 2,242 (83.8) | 1,882 (85.2) | 360 (77.3) |  |
|  | Yes | 434 (16.2) | 328 (14.8) | 106 (22.7) |  |
| Uric acid (mg/dL) |  | 5.41 (1.44) | 4.96 (1.05) | 7.53 (1.10) | <0.001^*^ |

*: Frequencies and percentages were tested using Fisher’s exact test, while continuous variables were compared using an independent t-test. *P*<0.05 was considered as significant.

Table S2. The comparison of intake rates and intake levels of various specific legumes between the HUA and non-HUA groups.

| Group | Non-HUA | HUA | *P* | Non-HUA  Mean (SD) | HUA  Mean (SD) | *P*^†^ |
| --- | --- | --- | --- | --- | --- | --- |
| Black, brown, or Bayo beans (cup eq.) |  |  | 0.617 | 0.020 (0.10) | 0.020 (0.10) | 0.587 |
| No | 94.04% | 94.89% |  |  |  |  |
| Yes | 5.96% | 5.11% |  |  |  |  |
| Chickpeas (oz. eq.) |  |  | 0.808 | 0.020 (0.17) | 0.020 (0.18) | 0.608 |
| No | 98.36% | 98.82% |  |  |  |  |
| Yes | 1.64% | 1.18% |  |  |  |  |
| Green peas (cup eq.) |  |  | 0.873 | 0.010 (0.07) | 0.010 (0.07) | 0.651 |
| No | 96.36% | 96.81% |  |  |  |  |
| Yes | 3.64% | 3.19% |  |  |  |  |
| Green string beans (cup eq.) |  |  | 0.733 | 0.050 (0.15) | 0.050 (0.17) | 0.775 |
| No | 87.40% | 88.13% |  |  |  |  |
| Yes | 12.60% | 11.87% |  |  |  |  |
| Immature lima beans (cup eq.) |  |  | 0.003^*^ | 0.002 (0.04) | 0.010 (0.07) | 0.025^§^ |
| No | 99.36% | 97.38% |  |  |  |  |
| Yes | 0.64% | 2.62% |  |  |  |  |
| Lentils (oz. eq.) |  |  | 0.671 | 0.030 (0.25) | 0.060 (0.46) | 0.551 |
| No | 98.05% | 97.66% |  |  |  |  |
| Yes | 1.95% | 2.34% |  |  |  |  |
| Peas, cowpeas, field peas, or blackeye peas (cup eq.) |  |  | 0.042^*^ | 0.001 (0.02) | 0.004 (0.05) | 0.155 |
| No | 99.87% | 99.11% |  |  |  |  |
| Yes | 0.13% | 0.89% |  |  |  |  |
| Pinto, calico, or red Mexican beans (oz. eq.) |  |  | 1 | 0.160 (0.62) | 0.200 (0.88) | 0.913 |
| No | 91.23% | 91.26% |  |  |  |  |
| Yes | 8.77% | 8.74% |  |  |  |  |
| Red kidney beans (oz. eq.) |  |  | 0.024^*^ | 0.060 (0.42) | 0.030 (0.30) | 0.005^§^ |
| No | 96.48% | 98.82% |  |  |  |  |
| Yes | 3.52% | 1.18% |  |  |  |  |
| Snowpea (cup eq.) |  |  | 1 | 0.001 (0.02) | 0.001 (0.01) | 0.874 |
| No | 99.62% | 99.70% |  |  |  |  |
| Yes | 0.38% | 0.30% |  |  |  |  |
| String beans (cup eq.) |  |  | 1 | 0.009 (0.07) | 0.008 (0.07) | 0.720 |
| No | 97.99% | 98.24% |  |  |  |  |
| Yes | 2.01% | 1.76% |  |  |  |  |
| White beans (cup eq.) |  |  | 0.378 | 0.004 (0.06) | 0.010 (0.11) | 0.387 |
| No | 99.05% | 98.53% |  |  |  |  |
| Yes | 0.95% | 1.47% |  |  |  |  |
| Yellow, canary, or Peruvian beans (oz. eq.) |  |  | 1 | 0.009 (0.16) | 0.018 (0.34) | 0.821 |
| No | 99.55% | 99.70% |  |  |  |  |
| Yes | 0.45% | 0.30% |  |  |  |  |

The Fisher’s exact test was used to compare the intake rates of various specific legumes between the HUA and non-HUA groups, while the t-test was employed to compare the intake levels of various specific legumes between the HUA and non-HUA groups. *: The difference was significant using Fisher’s exact test (two-sided, *P*<0.05); †: Data were log-transformed before t-test; §: The difference was significant using t-test (two-sided, *P*<0.05).

Table S3. Logistic regression analysis of the intake of various specific legumes and the risk of HUA.

| Group | N | Model 1 | | | Model 2 | | Model 3 | | |
| --- | --- | --- | --- | --- | --- | --- | --- | --- | --- |
|  |  | OR (95%CI) | *P* | OR (95%CI) | | *P* | | OR (95%CI) | *P* |
| Black, brown, or Bayo beans | 2,012 | 0.85 (0.51,1.42) | 0.536 | 0.85 (0.50,1.44) | | 0.542 | | 0.86 (0.51,1.45) | 0.568 |
| Chickpeas | 1,925 | 0.72 (0.25,2.07) | 0.542 | 0.78 (0.26,2.28) | | 0.645 | | 0.79 (0.27,2.31) | 0.661 |
| Green peas | 1,965 | 0.87 (0.45,1.68) | 0.680 | 0.74 (0.38,1.45) | | 0.381 | | 0.74 (0.38,1.45) | 0.383 |
| Green string beans | 2,165 | 0.93 (0.66,1.31) | 0.698 | 0.87 (0.61,1.23) | | 0.432 | | 0.87 (0.61,1.23) | 0.434 |
| Immature lima beans | 1,914 | 4.21 (1.70,10.43) | 0.002^*^ | 3.37 (1.31,8.70) | | 0.012^*^ | | 3.46 (1.33,8.97) | 0.011^*^ |
| Lentils | 1,934 | 1.21 (0.55,2.65) | 0.640 | 1.28 (0.57,2.89) | | 0.547 | | 1.30 (0.58,2.92) | 0.529 |
| Peas, cowpeas, field peas, or blackeye peas | 1,900 | 7.01 (1.17,42.12) | 0.033^*^ | 6.02 (0.97,37.17) | | 0.053 | | 5.71 (0.92,35.32) | 0.061 |
| Pinto, calico, or red Mexican beans | 2,077 | 0.997 (0.67,1.49) | 0.988 | 0.91 (0.60,1.39) | | 0.667 | | 0.91 (0.60,1.39) | 0.662 |
| Red kidney beans | 1,956 | 0.33 (0.12,0.91) | 0.032^*^ | 0.31 (0.11,0.88) | | 0.027^*^ | | 0.31 (0.11,0.88) | 0.028^*^ |
| Snowpea | 1,902 | 0.78 (0.09,6.49) | 0.817 | 0.78 (0.09,6.75) | | 0.821 | | 0.80 (0.09,6.93) | 0.839 |
| String beans | 1,933 | 0.88 (0.36,2.11) | 0.769 | 0.90 (0.36,2.23) | | 0.820 | | 0.88 (0.35,2.19) | 0.781 |
| White beans | 1,915 | 1.56 (0.56,4.32) | 0.394 | 1.30 (0.46,3.73) | | 0.621 | | 1.28 (0.45,3.67) | 0.648 |
| Yellow, canary, or Peruvian beans | 1,903 | 0.67 (0.08,5.44) | 0.706 | 0.64 (0.08,5.33) | | 0.680 | | 0.65 (0.08,5.46) | 0.696 |

Model 1: no adjustment factors included; Model 2: adjusted for gender, age, race, education, PIR, BMI, smoking, and alcohol use; Model 3: adjusted for diabetes and hypertension, based on model 2. *: *P*<0.05. Abbreviations: CI: confidence interval.

Table S4. Logistic regression analysis of the intake of various specific legumes and the risk of HUA across different age levels.

| Group | Age<55 | | | Age≥55 | | |
| --- | --- | --- | --- | --- | --- | --- |
|  | N | OR (95% CI) | *P* | N | OR (95% CI) | *P* |
| Black, brown, or Bayo beans | 1,325 | 0.73 (0.38,1.43) | 0.363 | 687 | 1.13 (0.45,2.83) | 0.799 |
| Chickpeas | 1,255 | 0.35 (0.05,2.70) | 0.315 | 670 | 1.44 (0.37,5.68) | 0.600 |
| Green peas | 1,268 | 1.14 (0.42,3.09) | 0.793 | 697 | 0.57 (0.23,1.42) | 0.229 |
| Green string beans | 1,392 | 0.92 (0.56,1.49) | 0.720 | 773 | 0.81 (0.49,1.35) | 0.418 |
| Immature lima beans | 1,239 | NA | 0.980 | 675 | 4.43 (1.57,12.49) | 0.005^*^ |
| Lentils | 1,263 | 1.91 (0.73,5.03) | 0.190 | 671 | 0.68 (0.14,3.24) | 0.632 |
| Peas, cowpeas, field peas, or blackeye peas | 1,238 | NA | 0.968 | 662 | 1.91 (0.16,23.60) | 0.613 |
| Pinto, calico, or red Mexican beans | 1,336 | 0.82 (0.43,1.54) | 0.534 | 741 | 1.02 (0.57,1.82) | 0.958 |
| Red kidney beans | 1,278 | 0.12 (0.02,0.91) | 0.040^*^ | 678 | 0.70 (0.20,2.48) | 0.579 |
| Snowpea | 1,238 | 8.81 (0.42,184.64) | 0.161 | 664 | NA | 0.982 |
| String beans | 1,254 | 0.84 (0.18,3.83) | 0.823 | 679 | 1.03 (0.33,3.26) | 0.956 |
| White beans | 1,246 | 0.60 (0.07,4.91) | 0.635 | 669 | 2.45 (0.65,9.19) | 0.185 |
| Yellow, canary, or Peruvian beans | 1,243 | NA | 0.979 | 660 | NA | 0.978 |

NA: Data for this categorization are not shown due to a too large confidence interval or an inestimable result. *: *P*<0.05. The logistic regression model used was stratified by age, and the covariate age was excluded in model 3.

Table S5. Logistic regression analysis of the intake of various specific legumes and the risk of HUA across different BMI levels.

| Group | Normal-BMI | | | Abnormal-BMI | | | Overweight | | | Obesity | | |
| --- | --- | --- | --- | --- | --- | --- | --- | --- | --- | --- | --- | --- |
|  | N | OR (95% CI) | *P* | N | OR (95% CI) | *P* | N | OR (95% CI) | *P* | N | OR (95% CI) | *P* |
| Black, brown, or Bayo beans | 534 | NA | 0.989 | 1,478 | 1.002 (0.58,1.72) | 0.994 | 624 | 0.67 (0.23,1.94) | 0.459 | 824 | 1.28 (0.66,2.49) | 0.463 |
| Chickpeas | 515 | 0.62 (0.06,6.12) | 0.679 | 1,410 | 0.71 (0.21,2.47) | 0.595 | 595 | 1.40 (0.29,6.65) | 0.675 | 786 | 0.48 (0.06,4.10) | 0.504 |
| Green peas | 523 | 0.43 (0.05,3.70) | 0.444 | 1,442 | 0.77 (0.38,1.57) | 0.469 | 606 | 0.49 (0.11,2.17) | 0.344 | 808 | 0.94 (0.40,2.18) | 0.882 |
| Green string beans | 570 | 0.44 (0.13,1.55) | 0.203 | 1,595 | 0.94 (0.65,1.36) | 0.741 | 664 | 0.86 (0.42,1.75) | 0.673 | 902 | 0.92 (0.59,1.43) | 0.711 |
| Immature lima beans | 509 | NA | 0.987 | 1,405 | 4.31 (1.55,11.95) | 0.005^A^ | 590 | 3.31 (0.72,15.26) | 0.125 | 787 | 6.58 (1.27,34.03) | 0.025^*^ |
| Lentils | 518 | 0.43 (0.05,3.98) | 0.460 | 1,416 | 1.48 (0.61,3.57) | 0.388 | 597 | 1.12 (0.24,5.15) | 0.889 | 789 | 3.04 (0.84,10.96) | 0.089 |
| Peas, cowpeas, field peas, or blackeye peas | 507 | NA | 0.989 | 1,393 | 9.75 (0.998,95.28) | 0.050 | 583 | NA | 0.981 | 782 | NA | 0.975 |
| Pinto, calico, or red Mexican beans | 535 | 0.39 (0.05,3.15) | 0.376 | 1,542 | 0.97 (0.63,1.50) | 0.900 | 636 | 0.72 (0.29,1.81) | 0.488 | 878 | 0.96 (0.58,1.61) | 0.890 |
| Red kidney beans | 519 | 0.49 (0.05,4.90) | 0.545 | 1,437 | 0.26 (0.08,0.84) | 0.025^A^ | 602 | 0.32 (0.04,2.50) | 0.278 | 807 | 0.23 (0.05,0.99) | 0.049^*^ |
| Snowpea | 509 | NA | 0.986 | 1,393 | 1.26 (0.13,12.41) | 0.846 | 585 | 4.42 (0.37,52.22) | 0.239 | 780 | NA | 0.981 |
| String beans | 521 | 0.56 (0.06,4.99) | 0.606 | 1,412 | 1.01 (0.36,2.81) | 0.987 | 592 | 0.80 (0.10,6.76) | 0.84 | 791 | 1.18 (0.34,4.11) | 0.800 |
| White beans | 509 | NA | 0.987 | 1,406 | 1.36 (0.46,3.98) | 0.578 | 589 | 1.63 (0.28,9.53) | 0.587 | 789 | 1.09 (0.27,4.36) | 0.904 |
| Yellow, canary, or Peruvian beans | 507 | NA | 0.990 | 1,396 | 0.67 (0.08,5.70) | 0.717 | 586 | NA | 0.985 | 782 | 1.15 (0.10,13.20) | 0.909 |

NA: Data for this categorization are not shown due to a too large confidence interval or an inestimable result. *: *P*<0.05. The logistic regression model used was stratified by BMI, and the covariate BMI was excluded in model 3.

Table S6. Logistic regression analysis of the intake of various specific legumes and the risk of HUA across different gender groups.

| Group | Male | | | Female | | |
| --- | --- | --- | --- | --- | --- | --- |
|  | N | OR (95% CI) | *P* | N | OR (95% CI) | *P* |
| Black, brown, or Bayo beans | 946 | 0.69 (0.31,1.50) | 0.343 | 1,066 | 0.96 (0.46,2.00) | 0.911 |
| Chickpeas | 903 | 1.40 (0.37,5.39) | 0.621 | 1,022 | 0.32 (0.04,2.51) | 0.280 |
| Green peas | 925 | 0.63 (0.24,1.67) | 0.352 | 1,040 | 0.89 (0.35,2.26) | 0.806 |
| Green string beans | 1,019 | 0.85 (0.52,1.39) | 0.520 | 1,146 | 0.88 (0.53,1.45) | 0.607 |
| Immature lima beans | 900 | 3.01 (0.80,11.34) | 0.103 | 1,014 | 3.98 (0.96,16.60) | 0.058 |
| Lentils | 910 | 1.70 (0.58,4.92) | 0.332 | 1,024 | 0.88 (0.24,3.18) | 0.845 |
| Peas, cowpeas, field peas, or blackeye peas | 892 | 5.11 (0.28,92.76) | 0.270 | 1,008 | 5.31 (0.46,61.89) | 0.183 |
| Pinto, calico, or red Mexican beans | 967 | 1.16 (0.64,2.11) | 0.626 | 1,110 | 0.74 (0.40,1.37) | 0.342 |
| Red kidney beans | 920 | 0.48 (0.14,1.62) | 0.235 | 1,036 | 0.15 (0.02,1.16) | 0.069 |
| Snowpea | 894 | 1.49 (0.14,15.71) | 0.740 | 1,008 | NA | 0.979 |
| String beans | 915 | 1.11 (0.40,3.11) | 0.835 | 1,018 | 0.60 (0.07,4.91) | 0.635 |
| White beans | 899 | 1.81 (0.43,7.53) | 0.416 | 1,016 | 0.96 (0.19,4.89) | 0.957 |
| Yellow, canary, or Peruvian beans | 893 | 1.82 (0.16,20.70) | 0.628 | 1,010 | NA | 0.982 |

NA: Data for this categorization are not shown due to a too large confidence interval or an inestimable result. The logistic regression model used was stratified according to gender, and the covariate gender was excluded in model 3.

Table S7. PLS-DA of legume intake and the prevalence risk of HUA in different groups.

| Group | VIP | | | | | | | | |
| --- | --- | --- | --- | --- | --- | --- | --- | --- | --- |
|  | Overall  (N=2,676) | Age≥55  (N=983) | Age<55  (N=1,693) | Male  (N=1,268) | Female  (N=1,408) | Normal-BMI  (N=681) | Abnormal-BMI  (N=1,995) | Overweight  (N=838) | Obesity  (N=1,123) |
| Black, brown, or Bayo beans | 0.42 | 0.26 | 0.42 | 1.27 | 0.24 | 2.51 | 0.13 | 0.93 | 0.45 |
| Chickpeas | 0.43 | 0.38 | 0.83 | 0.31 | 0.77 | 0.86 | 0.52 | 0.21 | 0.53 |
| Green peas | 0.27 | 0.82 | 0.14 | 0.99 | 0.26 | 0.29 | 0.27 | 0.91 | 0.13 |
| Green string beans | 0.26 | 0.71 | 0.12 | 0.78 | 0.23 | 1.26 | 0.20 | 0.59 | 0.29 |
| Immature lima beans | 2.45 | 2.58 | 0.54 | 1.80 | 2.45 | 0.83 | 2.37 | 2.17 | 2.09 |
| Lentils | 0.39 | 0.39 | 0.86 | 0.63 | 0.26 | 0.38 | 0.45 | 0.14 | 1.13 |
| Peas, cowpeas, field peas, or blackeye peas | 1.78 | 0.35 | 2.52 | 1.20 | 1.80 | 0.47 | 1.81 | 0.45 | 1.94 |
| Pinto, calico, or red Mexican beans | 0.05 | 0.11 | 0.36 | 0.51 | 0.17 | 1.29 | 0.16 | 0.67 | 0.25 |
| Red kidney beans | 1.60 | 0.57 | 1.64 | 1.59 | 1.33 | 0.20 | 1.73 | 1.30 | 1.61 |
| Snowpea | 0.16 | 0.96 | 1.05 | 0.28 | 0.52 | 0.81 | 0.13 | 1.09 | 0.41 |
| String beans | 0.19 | 0.23 | 0.30 | 0.07 | 0.54 | 0.09 | 0.08 | 0.40 | 0.36 |
| White beans | 0.67 | 1.11 | 0.31 | 1.14 | 0.29 | 0.81 | 0.62 | 1.30 | 0.24 |
| Yellow, canary, or Peruvian beans | 0.26 | 1.47 | 0.80 | 0.65 | 0.67 | 0.47 | 0.30 | 0.90 | 0.17 |

Table S8. Sensitivity analysis of logistic regression analysis on the intake of key legumes and the risk of HUA among the overall population.

| Group | N | *P** | OR (95%CI) |
| --- | --- | --- | --- |
| Red kidney beans | 1,956 | 0.027 | 0.31 (0.11, 0.88) |
| Immature lima beans | 1,914 | 0.012 | 3.40 (1.32, 8.84) |

*: adjusted for gender, age, race, education, PIR, BMI, smoking, alcohol use, diabetes, hypertension, and sample weights.

Table S9. Dose-response relationship between levels of intake of key legumes and the risk of HUA among the overall population.

| Group | N | *P** | OR (95%CI) |
| --- | --- | --- | --- |
| Red kidney beans | 1,956 | 0.035 | 0.71 (0.52, 0.98) |
| Immature lima beans | 1,914 | 0.009 | 1.65 (1.13, 2.41) |

*: adjusted for gender, age, race, education, PIR, BMI, smoking, alcohol use, diabetes, and hypertension.

Table S10. Targets of HUA-related pathways overlapped with those of 5-MTHF..

| Metabolism | Targets for 5-MTHF |
| --- | --- |
| Alanine, aspartate and glutamate metabolism | AGXT |
| Arachidonic acid metabolism | PLA2G2A |
| Arginine and proline metabolism | NOS2 |
| Arginine biosynthesis | NOS2 |
| Arginine biosynthesis | OTC |
| Carbon fixation in photosynthetic organisms | ALDOA |
| Carbon fixation in photosynthetic organisms | TPI1 |
| Drug metabolism - other enzymes | CDA |
| Drug metabolism - other enzymes | IMPDH2 |
| Drug metabolism - other enzymes | TK1 |
| Galactose metabolism | AGXT |
| Glycolysis / Gluconeogenesis | ADH5 |
| Glycolysis / Gluconeogenesis | ALDOA |
| Glycolysis / Gluconeogenesis | PCK1 |
| Glycolysis / Gluconeogenesis | PDHB |
| Glycolysis / Gluconeogenesis | TPI1 |
| Glyoxylate and dicarboxylate metabolism | AGXT |
| Nicotinate and nicotinamide metabolism | NMNAT1 |
| Pentose phosphate pathway | ALDOA |
| Purine metabolism | IMPDH2 |
| Purine metabolism | PDE4D |
| Starch and sucrose metabolism | PYGL |
| Tryptophan metabolism | CAT |
| Tryptophan metabolism | KYAT1 |

Table S11. The targets of HUA-related pathways overlapped with those of anthocyanins.

| Metabolism | Targets for anthocyanins |
| --- | --- |
| Arachidonic acid metabolism | PTGS2 |
| Caffeine metabolism | XDH(XO) |
| Drug metabolism - other enzymes | TYMP |
| Drug metabolism - other enzymes | UCK2 |
| Drug metabolism - other enzymes | XDH |
| Galactose Metabolism | AKR1B1 |
| Galactose Metabolism | HK1 |
| Glutathione metabolism | GSR |
| Glycolysis / Gluconeogenesis | ADH1B |
| Glycolysis / Gluconeogenesis | HK1 |
| Glycolysis / Gluconeogenesis | PCK1 |
| Nicotinate and nicotinamide metabolism | CD38 |
| Purine metabolism | XDH(XO) |
| Starch and sucrose metabolism | AMY1A |
| Starch and sucrose metabolism | HK1 |
| Starch and sucrose metabolism | PYGL |

Table S12. Docking score for 5-MTHF to potential targets.

| Targets for 5-MTHF | PDB ID | Docking score |
| --- | --- | --- |
| ADH5 | 1M6W | -10.432 |
| AGXT | 1H0C | -5.355 |
| ALDOA | 1ALD | -6.794 |
| CAT | 1DGB | -7.971 |
| CDA | 1MQ0 | -9.46 |
| GALE | 1EK5 | -8.317 |
| IMPDH2 | 1B3O | -7.217 |
| KYAT1 | 1W7L | -3.43 |
| NMNAT1 | 1GZU | -5.954 |
| NOS2 | 1NSI | -6.952 |
| OTC | 1C9Y | -6.673 |
| PCK1 | 1KHB | -9.641 |
| PDE4D | 1XOM | -9.62 |
| PDHB | 1KHB | -9.392 |
| PLA2G2A | 1AYP | -10.107 |
| PYGL | 1EM6 | -7.934 |
| TK1 | 1W4R | -3.963 |
| TPI1 | 1HTI | -5.395 |

Table S13. Docking score for anthocyanins to potential targets.

| Targets for anthocyanins | PDB ID |  | Docking score |
| --- | --- | --- | --- |
| XDH | 2CKJ |  |  |
|  |  | Cy-3G | -7.563 |
|  |  | Dp-3G | -11.453 |
| AKR1B1 | 1ADS |  |  |
|  |  | Cy-3G | -2.724 |
|  |  | Dp-3G | -5.637 |
| AMY1A | 1C8Q |  |  |
|  |  | Cy-3G | -6.679 |
|  |  | Dp-3G | -5.096 |
| ADH1B | 1DEH |  |  |
|  |  | Cy-3G | -8.835 |
|  |  | Dp-3G | -10.591 |
| GSR | 1DNC |  |  |
|  |  | Cy-3G | -7.123 |
|  |  | Dp-3G | -9.19 |
| UCK2 | 1UJ2 |  |  |
|  |  | Cy-3G | -6.144 |
|  |  | Dp-3G | -5.701 |
| CD38 | 2I65 |  |  |
|  |  | Cy-3G | -9.98 |
|  |  | Dp-3G | -10.263 |
| PCK1 | 1KHB |  |  |
|  |  | Dp-3G | -9.816 |
|  |  | Cy-3G | -7.42 |
| PYGL | 1EM6 |  |  |
|  |  | Dp-3G | -10.855 |
|  |  | Cy-3G | -9.292 |
| PTGS2 | 5F19 |  |  |
|  |  | Cy-3G | -9.84 |
|  |  | Dp-3G | -6.487 |
| TYMP | 1UOU |  |  |
|  |  | Dp-3G | -6.112 |
|  |  | Cy-3G | -5.73 |
| HK1 | 1CZA |  |  |
|  |  | Cy-3G | -8.854 |
|  |  | Dp-3G | -8.03 |

Table S14. Pathway analysis of differential metabolites between the HUA model and Dp-3G-treated groups.

|  | Match Status | *P* | -log(*P*) |
| --- | --- | --- | --- |
| Cysteine and methionine metabolism | 3/33 | 0.009 | 2.028 |
| Purine metabolism | 4/71 | 0.014 | 1.851 |
| Tryptophan metabolism | 3/41 | 0.017 | 1.768 |
| Glyoxylate and dicarboxylate metabolism | 2/32 | 0.070 | 1.156 |
| Sphingolipid metabolism | 2/32 | 0.070 | 1.156 |
| Nitrogen metabolism | 1/6 | 0.080 | 1.098 |
| Arginine and proline metabolism | 2/36 | 0.086 | 1.067 |
| Steroid hormone biosynthesis | 3/79 | 0.091 | 1.043 |
| Pyrimidine metabolism | 2/39 | 0.098 | 1.007 |
| One carbon pool by folate | 1/9 | 0.117 | 0.930 |
| Biotin metabolism | 1/10 | 0.130 | 0.887 |
| Arginine biosynthesis | 1/14 | 0.177 | 0.752 |
| D-Amino acid metabolism | 1/15 | 0.188 | 0.725 |
| Butanoate metabolism | 1/15 | 0.188 | 0.725 |
| Folate biosynthesis | 1/27 | 0.314 | 0.503 |
| Alanine, aspartate and glutamate metabolism | 1/28 | 0.324 | 0.490 |
| Glycine, serine and threonine metabolism | 1/34 | 0.379 | 0.422 |
| Tyrosine metabolism | 1/42 | 0.445 | 0.351 |

Table S15. Enrichment analysis of differential metabolites between the HUA model and Dp-3G-treated groups.

|  | Match Status | *P* | -log(*P*) |
| --- | --- | --- | --- |
| Cysteine and methionine metabolism | 3/33 | 0.009 | 2.037 |
| Purine metabolism | 4/70 | 0.013 | 1.883 |
| Tryptophan metabolism | 3/41 | 0.017 | 1.777 |
| Glyoxylate and dicarboxylate metabolism | 2/31 | 0.065 | 1.187 |
| Sphingolipid metabolism | 2/32 | 0.069 | 1.162 |
| Nitrogen metabolism | 1/6 | 0.079 | 1.101 |
| Arginine and proline metabolism | 2/36 | 0.085 | 1.073 |
| Pyrimidine metabolism | 2/39 | 0.097 | 1.013 |
| Steroid hormone biosynthesis | 3/87 | 0.111 | 0.955 |
| One carbon pool by folate | 1/9 | 0.117 | 0.932 |
| Biotin metabolism | 1/10 | 0.129 | 0.889 |
| Arginine biosynthesis | 1/14 | 0.176 | 0.754 |
| D-Amino acid metabolism | 1/15 | 0.187 | 0.728 |
| Butanoate metabolism | 1/15 | 0.187 | 0.728 |
| Folate biosynthesis | 1/26 | 0.302 | 0.520 |
| Alanine, aspartate and glutamate metabolism | 1/28 | 0.322 | 0.492 |
| Glycine, serine and threonine metabolism | 1/33 | 0.368 | 0.434 |
| Tyrosine metabolism | 1/42 | 0.443 | 0.354 |

Table S16. Implications of Dp-3G and AP-altered metabolites in diseases related to HUA.

| Metabolite | AP-treated group | Dp-3G-treated group | Relationship with hyperuricemia-related diseases | Human | Animal | Cell | Mechanism |
| --- | --- | --- | --- | --- | --- | --- | --- |
| Methionine | Down^*^ | Down^*^ | Atherosclerosis | - The severity of atherosclerotic disease in young patients was associated with high post-methionine-induced metabolic disturbance [1]. | - Mice fed methionine-rich diets exhibited significant atheromatous pathology in the aortic arch [2]. - Dietary supplementation with methionine promoted early atherosclerosis in ApoE-deficient mice [3]. - Histological examination of the aorta in methionine-fed rabbits revealed typical atherosclerotic changes [4]. - In the rabbit experiment, endothelial cells in the methionine-fed group showed significant dysfunction related to atherosclerosis [5]. | - The disruption of methionine metabolism promoted the proliferation of vascular smooth muscle cells in a dose-dependent manner, potentially leading to their excessive growth, which is a critical risk factor for atherosclerosis [6]. - Methionine exposure disrupted endothelial barrier function associated with atherosclerosis in endothelial cells [7]. | - Methionine-induced metabolic disturbance promoted the proliferation of arterial smooth muscle cells and enhanced collagen synthesis and accumulation [3]. - The disturbance of lipid peroxidation and antioxidant processes may underlie the atherosclerotic effects of methionine [4]. - Vascular endothelial dysfunction played a significant role in the development of atherosclerosis following excessive methionine intake [5]. - Excessive dietary methionine may induce atherosclerosis by elevating plasma lipid levels and/or contributing to endothelial cell injury or dysfunction [7]. |
|  |  |  | Hypertension | - A cohort study of patients with cardiovascular disease found a positive association between methionine intake and higher blood pressure [8]. | - A methionine-enriched diet induced a significant increase of systolic blood pressure and Angiotensin II -induced contractile response in the aortae of rats [9]. | - Disruption of methionine metabolism led to increased endothelin type B receptor-mediated contraction and upregulation of endothelin type B receptor expression in rat vascular smooth muscle cells [10]. | - A methionine-enriched diet may affect the activation of Angiotensin-converting enzyme, potentially exacerbating the pathological condition of hypertension [9]. - Disruption of methionine metabolism may activate the ERK1/2 signaling pathway and NF-κB, thereby upregulating endothelin type B receptors in vascular smooth muscle cells [10]. - Methionine can inhibit the synthesis of nitric oxide, which leads to increased blood pressure [11]. |
| Proline | Down^*^ | Down^*^ | Hypertension | - A dietary pattern rich in proline-rich amino acids was associated with an increased risk of hypertension [12]. - High dietary intakes of proline may increase the risk of developing hypertension [13]. | - Microinjection of proline into the third ventricle or paraventricular nucleus of the hypothalamus in unanesthetized rats caused dose-related pressor effects [14]. | - The absorption of proline contributed to arterial remodeling in cultured rat vascular smooth muscle cells in the context of hypertension [15]. | - The pressor response to microinjection of proline was found to be mediated by circulating vasopressin [14]. - Increased uptake of proline promoted intracellular levels necessary for collagen synthesis and cell growth, thus promoting arterial remodeling in hypertension [15]. |
| Folate | Up^†^ | Up^*^ | Chronic kidney disease | - Folic acid treatment was associated with a greater reduction in the odds of CKD progression among patients with mild to moderate CKD [16]. - A 30-year follow-up study demonstrated a longitudinal association between higher folate intake in youth and a lower incidence of CKD in adulthood [17]. - Enalapril-folic acid therapy, compared to enalapril alone, can significantly delay the progression of CKD among patients with mild-to-moderate CKD [18]. | - Long-term dietary folate deficiency exacerbated renal fibrosis in mice by enhancing kidney-focused inflammation [19]. | - 5-MTHF can reduce hypoxia-reoxygenation-induced oxidative stress and alleviate damage to human kidney proximal tubular cells [20]. | - Poor dietary folate status may exacerbate renal dysfunction and inflammation in mice by enhancing systemic inflammation [19]. - Low-dose folic acid may improve renal function by activating Nrf2 and restoring antioxidant defenses [20]. |
|  |  |  | Atherosclerosis | - Lower levels of folate were associated with an increased risk of atherosclerosis [21]. - Low folate levels may be an atherogenic factor in young, healthy nonsmokers [22]. | - Supplementation of folate in an atherogenic diet inhibited the development of atherosclerotic lesions in mice [23]. | - Experiments using the human EA. hy926 endothelial cell line showed that mild folic acid deficiency can cause atherosclerosis [24]. | - Folic acid supplementation delayed the development of atherosclerotic lesions by modulating monocyte chemoattractant protein 1 and vascular endothelial growth factor DNA methylation levels both in vivo and in vitro [23]. |
|  |  |  | Hypertension | - Higher total folate intake was associated with a decreased risk of incident hypertension, particularly in younger women [25]. - Folic acid supplementation was effective in the primary prevention of reducing blood pressure among patients with hypertension and hyperhomocysteinemia [26]. - Higher folate intake in young adulthood was associated with a lower incidence of hypertension later in life [27]. - Short-term folic acid supplementation significantly reduced blood pressure in young chronic smokers [28]. | - Folic acid prevented and partially reversed glucocorticoid-induced hypertension in rats [29]. | - Folic acid can restore NO metabolism in human pulmonary artery endothelial cells under hypoxic conditions, thereby alleviating the progression of pulmonary hypertension [30]. | - Supplemental folic acid improved endothelial function in vivo [25]. - Folic acid can restore the reduced level of dihydrofolate reductase in human pulmonary artery endothelial cells under hypoxic conditions, thereby maintaining the balance between tetrahydrobiopterin-7,8-dihydrobiopterin. This helps prevent eNOS uncoupling, improves nitric oxide bioavailability, and may slow the progression of pulmonary hypertension [30]. |

*: the difference was significant (*P*<0.05); †: the difference had a trend but insignificant.

**Supplemental Movie**


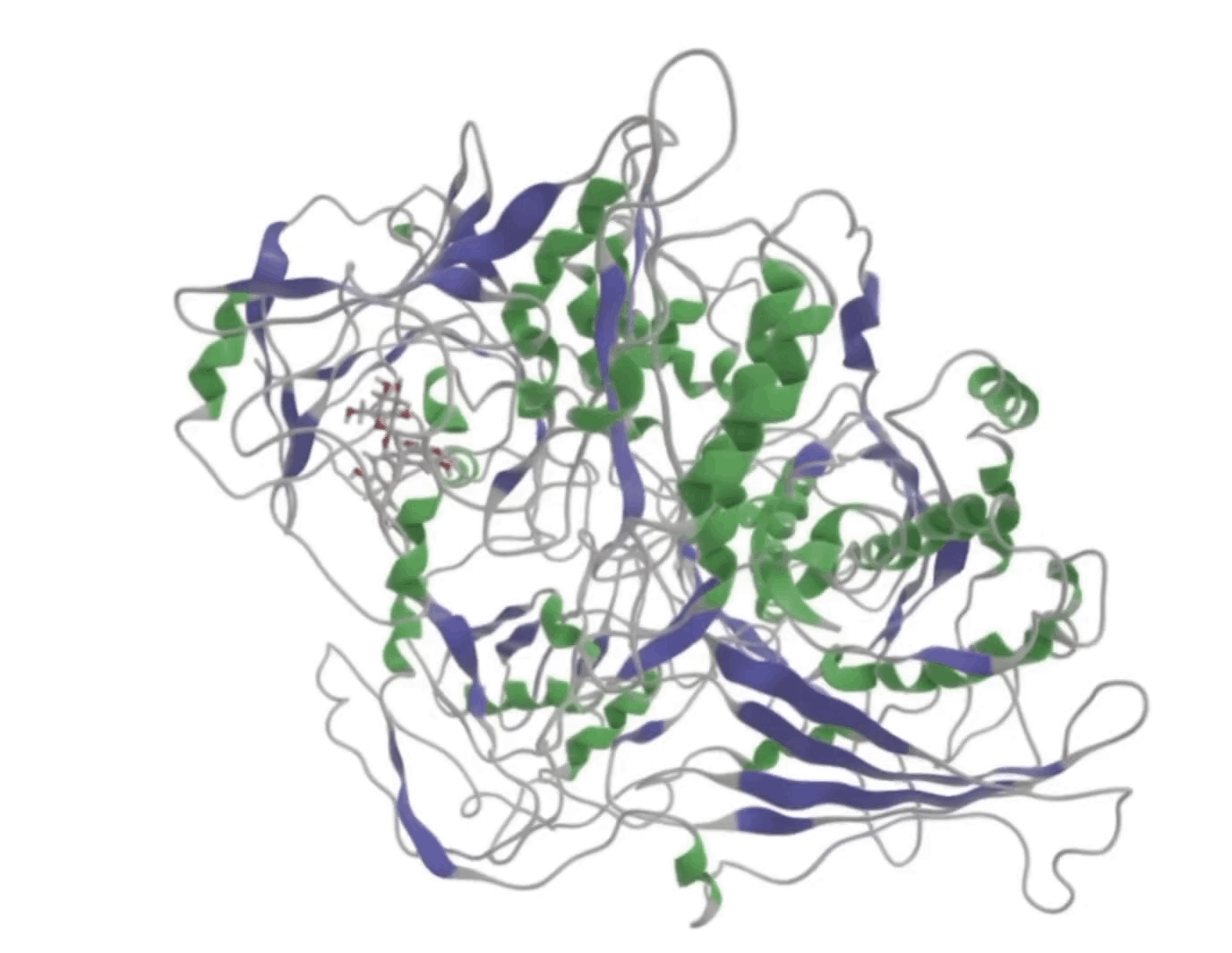


Movie S1. Molecular dynamics simulations of the binding between Dp-3G and the XO enzyme. (separate file)

**Supplemental Data**

**Data S1.** Metabolomics data. **(separate file)**

**References**

1. van den Berg M, Stehouwer CD, Bierdrager E, Rauwerda JA. Plasma homocysteine and severity of atherosclerosis in young patients with lower-limb atherosclerotic disease. Arterioscler Thromb Vasc Biol. 1996;16(1):165-71.

2. Troen AM, Lutgens E, Smith DE, Rosenberg IH, Selhub J. The atherogenic effect of excess methionine intake. Proceedings of the National Academy of Sciences of the United States of America. 2003;100(25):15089-94.

3. Zhou J, Møller J, Danielsen CC, Bentzon J, Ravn HB, Austin RC, et al. Dietary supplementation with methionine and homocysteine promotes early atherosclerosis but not plaque rupture in ApoE-deficient mice. Arterioscler Thromb Vasc Biol. 2001;21(9):1470-6.

4. Toborek M, Kopieczna-Grzebieniak E, Drózdz M, Wieczorek M. Increased lipid peroxidation as a mechanism of methionine-induced atherosclerosis in rabbits. Atherosclerosis. 1995;115(2):217-24.

5. Fujimoto S, Togane Y, Matsuzaki C, Yamashina S, Nakano H, Yamazaki J, et al. Effects of long-term administration of methionine on vascular endothelium in rabbits. Nutr Metab Cardiovasc Dis. 2003;13(1):20-7.

6. Zhan XL, Yang XH, Gu YH, Guo LL, Jin HM. Epigallocatechin gallate protects against homocysteine-induced vascular smooth muscle cell proliferation. Mol Cell Biochem. 2018;439(1-2):131-40.

7. Toborek M, Hennig B. Dietary methionine imbalance, endothelial cell dysfunction and atherosclerosis. Nutrition Research. 1996;16(7):1251-66.

8. Tuttle KR, Milton JE, Packard DP, Shuler LA, Short RA. Dietary amino acids and blood pressure: a cohort study of patients with cardiovascular disease. Am J Kidney Dis. 2012;59(6):803-9.

9. Zhou Y, Zhao L, Zhang Z, Lu X. Protective Effect of Enalapril against Methionine-Enriched Diet-Induced Hypertension: Role of Endoplasmic Reticulum and Oxidative Stress. Biomed Res Int. 2015;2015:724876.

10. Chen Y, Zhang H, Liu E, Xu CB, Zhang Y. Homocysteine regulates endothelin type B receptors in vascular smooth muscle cells. Vascul Pharmacol. 2016;87:100-9.

11. Poggiogalle E, Fontana M, Giusti AM, Pinto A, Iannucci G, Lenzi A, et al. Amino Acids and Hypertension in Adults. Nutrients. 2019;11(7).

12. Teymoori F, Asghari G, Mirmiran P, Azizi F. Dietary amino acids and incidence of hypertension: A principle component analysis approach. Scientific reports. 2017;7(1):16838.

13. Teymoori F, Asghari G, Farhadnejad H, Nazarzadeh M, Atifeh M, Mirmiran P, et al. Various proline food sources and blood pressure: substitution analysis. Int J Food Sci Nutr. 2020;71(3):332-40.

14. Lopes-Azevedo S, Scopinho AA, Busnardo C, Aguiar Corrêa FM. Cardiovascular effects of the microinjection of L-proline into the third ventricle or the paraventricular nucleus of the hypothalamus in unanesthetized rats. J Neurosci Res. 2012;90(11):2183-92.

15. Reyna SV, Ensenat D, Johnson FK, Wang H, Schafer AI, Durante W. Cyclic strain stimulates L-proline transport in vascular smooth muscle cells. Am J Hypertens. 2004;17(8):712-7.

16. Huang S, Mu F, Li F, Wang W, Chen H, Lei L, et al. A Network‐Based Approach to Explore the Mechanism and Bioactive Compounds of Erzhi Pill against Metabolic Dysfunction‐Associated Fatty Liver Disease. 2020;2020(1):7867245.

17. Liu M, Ye Z, Wu Q, Yang S, Zhang Y, Zhou C, et al. Folate intake and incident chronic kidney disease: a 30-year follow-up study from young adulthood to midlife. The American journal of clinical nutrition. 2022;116(2):599-607.

18. Xu X, Qin X, Li Y, Sun D, Wang J, Liang M, et al. Efficacy of Folic Acid Therapy on the Progression of Chronic Kidney Disease: The Renal Substudy of the China Stroke Primary Prevention Trial. JAMA Intern Med. 2016;176(10):1443-50.

19. Chan CW, Lin BF. Folate Deficiency Enhanced Inflammation and Exacerbated Renal Fibrosis in High-Fat High-Fructose Diet-Fed Mice. Nutrients. 2023;15(16).

20. Wijerathne CUB, Au-Yeung KKW, Siow YL, O K. 5-Methyltetrahydrofolate Attenuates Oxidative Stress and Improves Kidney Function in Acute Kidney Injury through Activation of Nrf2 and Antioxidant Defense. Antioxidants (Basel, Switzerland). 2022;11(6).

21. Robinson K, Arheart K, Refsum H, Brattström L, Boers G, Ueland P, et al. Low circulating folate and vitamin B6 concentrations: risk factors for stroke, peripheral vascular disease, and coronary artery disease. European COMAC Group. Circulation. 1998;97(5):437-43.

22. Imamura A, Murakami R, Takahashi R, Cheng XW, Numaguchi Y, Murohara T, et al. Low folate levels may be an atherogenic factor regardless of homocysteine levels in young healthy nonsmokers. Metabolism: clinical and experimental. 2010;59(5):728-33.

23. Cui S, Li W, Lv X, Wang P, Gao Y, Huang G. Folic Acid Supplementation Delays Atherosclerotic Lesion Development by Modulating MCP1 and VEGF DNA Methylation Levels In Vivo and In Vitro. International journal of molecular sciences. 2017;18(5).

24. Brown KS, Huang Y, Lu ZY, Jian W, Blair IA, Whitehead AS. Mild folate deficiency induces a proatherosclerotic phenotype in endothelial cells. Atherosclerosis. 2006;189(1):133-41.

25. Forman JP, Rimm EB, Stampfer MJ, Curhan GC. Folate intake and the risk of incident hypertension among US women. Jama. 2005;293(3):320-9.

26. Wang WW, Wang XS, Zhang ZR, He JC, Xie CL. A Meta-Analysis of Folic Acid in Combination with Anti-Hypertension Drugs in Patients with Hypertension and Hyperhomocysteinemia. Frontiers in pharmacology. 2017;8:585.

27. Xun P, Liu K, Loria CM, Bujnowski D, Shikany JM, Schreiner PJ, et al. Folate intake and incidence of hypertension among American young adults: a 20-y follow-up study. The American journal of clinical nutrition. 2012;95(5):1023-30.

28. Mangoni AA, Sherwood RA, Swift CG, Jackson SH. Folic acid enhances endothelial function and reduces blood pressure in smokers: a randomized controlled trial. J Intern Med. 2002;252(6):497-503.

29. Miao Y, Zhang Y, Lim PS, Kanjanapan Y, Mori TA, Croft KD, et al. Folic acid prevents and partially reverses glucocorticoid-induced hypertension in the rat. Am J Hypertens. 2007;20(3):304-10.

30. Chalupsky K, Kračun D, Kanchev I, Bertram K, Görlach A. Folic Acid Promotes Recycling of Tetrahydrobiopterin and Protects Against Hypoxia-Induced Pulmonary Hypertension by Recoupling Endothelial Nitric Oxide Synthase. Antioxid Redox Signal. 2015;23(14):1076-91.
